# Identification and quantification of biosurfactants produced by the marine bacterium *Alcanivorax borkumensis* by hyphenated techniques

**DOI:** 10.1007/s00216-023-04972-5

**Published:** 2023-10-11

**Authors:** Anna Lipphardt, Tobias Karmainski, Lars M. Blank, Heiko Hayen, Till Tiso

**Affiliations:** 1 Institute of Inorganic and Analytical Chemistry, University of Münster, Münster, Germany; 2 Institute of Applied Microbiology, RWTH Aachen University, Aachen, Germany

**Keywords:** Glucolipid, Hydrocarbonoclastic bacteria, HPLC, Charged-aerosol detection, Mass spectrometry

## Abstract

A novel biosurfactant was discovered to be synthesized by the marine bacterium *Alcanivorax borkumensis* in 1992. This bacterium is abundant in marine environments affected by oil spills, where it helps to degrade alkanes and, under such conditions, produces a glycine-glucolipid biosurfactant. The biosurfactant enhances the bacterium’s attachment to oil droplets and facilitates the uptake of hydrocarbons. Due to its useful properties expected, there is interest in the biotechnological production of this biosurfactant. To support this effort analytically, a method combining reversed-phase high-performance liquid chromatography (HPLC) with high-resolution mass spectrometry (HRMS) was developed, allowing the separation and identification of glycine-glucolipid congeners. Accurate mass, retention time, and characteristic fragmentation pattern were utilized for species assignment. In addition, charged-aerosol detection (CAD) was employed to enable absolute quantification without authentic standards. The methodology was used to investigate the glycine-glucolipid production by *A. borkumensis* SK2 using different carbon sources. Mass spectrometry allowed us to identify congeners with varying chain lengths (C_6_–C_12_) and degrees of unsaturation (0–1 double bonds) in the incorporated 3-hydroxy-alkanoic acids, some previously unknown. Quantification using CAD revealed that the titer was approximately twice as high when grown with hexadecane as with pyruvate (49 mg/L *versus* 22 mg/L). The main congener for both carbon sources was glc-40:0-gly, accounting for 64% with pyruvate and 85% with hexadecane as sole carbon source. With the here presented analytical suit, complex and varying glycolipids can be identified, characterized, and quantified, as here exemplarily shown for the interesting glycine-glucolipid of *A. borkumensis.*

**Graphical Abstract:**

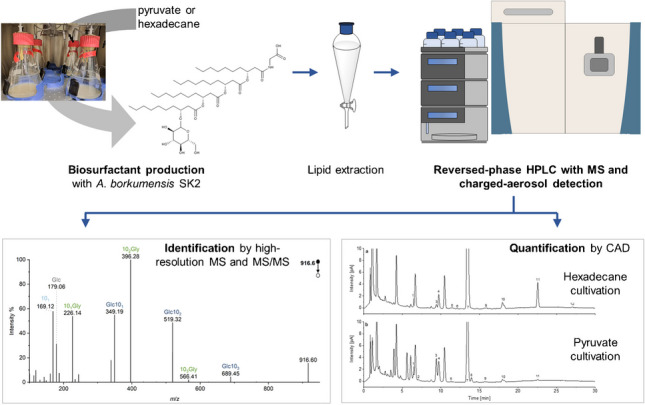

**Supplementary Information:**

The online version contains supplementary material available at 10.1007/s00216-023-04972-5.

## Introduction

In 1992, Passeri et al*.* [1] first described the production of a novel biosurfactant by the marine bacterium *Alcanivorax borkumensis* isolated from North Sea water and sediment samples, more precisely, from waters near the island of Borkum, which accounts for its name. The hydrocarbonoclastic, *i.e.*, oil-degrading, bacterium produces a cell wall-associated glycine-glucolipid to enhance cell attachment to oil droplets, facilitating hydrocarbon uptake [2–4]. Notably, *A. borkumensis* is one of the most abundant marine bacteria in waters and coastlines of oil spills, where it degrades alkanes, comprising up to 80–90% of the oil-degrading microbial community [5]. At the same time, it is very rarely found in unpolluted marine environments. Bacteria belonging to this genus encompass the ability to use a wide range of hydrocarbons as sole carbon and energy sources [6, 7] and thus belong to the obligate hydrocarbonoclastic bacteria (OHCB) [8]. In addition to evaporation and weathering processes, microbial oil degradation contributes to the natural removal of pollutants. It is, therefore, of particular relevance concerning the ongoing pollution by crude oil-derived hydrocarbons due to human activities.

**Fig. 1:**
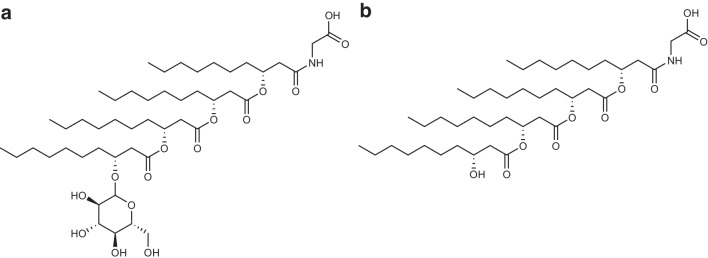
Chemical structure of the glycine-glucolipid from *A. borkumensis* using the example of **a** the main congener glc-10:04-gly and **b** the corresponding aglycone 10:04-gly

The surface-active glycolipid formed was first identified as anionic glucose lipid, consisting of glucose attached to a lipid backbone of four 3-hydroxy-fatty acids [1]. Later, Abraham et al. [9] found that the glucolipids are linked to glycine in amide linkage at the carboxyl group of the terminal fatty acid and fully elucidated the structure of the glucosyl-tetra(3-hydroxy-acyl)-glycine (Fig. 1). It was speculated that a glycine-containing form and a glycine-free form exist and the glycine-free form might be secreted into the culture supernatant in contrast to the glycine-containing form being associated to the cell wall [4]. However, recent studies do not provide evidence for glycine-free congeners [10].

With the new findings on the structure of the produced biosurfactant, Abraham et al*.* [9] also reported other derivatives of this lipid type identified in *A. borkumensis* in 1998. Their studies by sector field tandem mass spectrometry (MS) after ionization *via* fast atom bombardment and nuclear magnetic resonance (NMR) spectroscopy resulted in ten congeners of this anionic glycine-glucolipid with a tetrameric oxyacyl side chain. The ten identified congeners differ by the chain length of one or two of the four linked 3-hydroxy-fatty acids (C_6_, C_8_, and C_10_).

As this biosurfactant, like other glycolipids, is expected to be of interest for various industrial applications (*e.g.*, soaps and detergents, food products, cosmetics, agriculture, pharmacology, oil recovery, and bioremediation) due to its amphiphilic properties [11], targeted biotechnological production on a larger scale is of interest. As with glycolipids already on the market, *e.g.*, sophorolipids and rhamnolipids, an enormous commercial potential (biosurfactants market revenue of 1.2 billion USD in 2022, forecast revenue of 1.9 billion USD in 2027 [12]) can be assumed due to the expected biodegradability and low eco-toxicity as well as the carbon-neutral biotechnological production based on various renewable feedstocks. In addition, newly engineered structures possess valuable new properties [10]. These factors make their future use as an alternative or supplement to petroleum-based surfactants a promising proposition [10]. Therefore, powerful analytical techniques accompanying the fermentation process are needed to help solve the challenges in microbial production and improve yields and reduce costs.

So far, several analytical techniques have been applied to study glycine-glucolipids from *A. borkumensis*. For complete elucidation of the detailed structure of this lipid type, 1D- and 2D-NMR analysis was performed in the 1990s [1, 9]. The results were supported by the use of fast atom bombardment sector field MS in positive and negative ionization modes [1, 9] including tandem mass spectrometry in negative ion mode [9]. In addition, partial structures were confirmed by HPLC-diode array detection (DAD) and gas chromatography (GC)-MS, both requiring additional sample preparation, including acid hydrolysis for characterization of the sugar head group by HPLC-DAD [1] or hydrolysis, methylation, and trimethylsilylation for analysis of 3-hydroxy-fatty acids as methyl esters by GC-MS [1, 10].

As the common structure is known, additional congeners were detected by direct infusion nano electrospray-ionization quadrupole time-of-flight (QTOF)-MS and confirmed *via* fragmentation experiments (MS/MS) [10]. However, direct infusion MS suffers several limitations when analyzing complex samples, especially when the analyte of interest is not the major constituent of a sample or when additional glycine-glucolipid congeners are present at low levels [1, 9, 10]. Online hyphenation of HPLC and electrospray ionization-MS is expected to provide a more comprehensive analysis of the complex samples. Chromatographic separation of matrix components, isobaric interferences, and other interfering compounds facilitates the identification and increases sensitivity for minor components. Online hyphenation of HPLC and MS for the glycine-glucolipid from *A. borkumensis* has not yet been published, although it is desirable to remedy difficulties and uncertainties in identification. The established application of HPLC-MS for identification of other biosurfactants, *i.e.*, liamocins (polyol lipids) [13], 3-(3-hydroxyalkanoyloxy)alkanoic acids (HAA [14]), rhamnolipids [14–17], sophorolipids [18], and mannosylerythritol lipids [19], demonstrates the necessity and relevance of this analytical approach.

In addition, a simple and rapid technique for the quantification of glycine-glucolipids is needed to foster the biotechnological development of these promising glycolipids. Until now, a method based on reversed-phase HPLC-DAD has been published for quantification after the conversion of the glycine-glucolipid congeners devoid of strong chromophores into its phenacyl esters [10]. Since DAD does not yield structural information, the signals in the HPLC-DAD chromatogram still must be assigned separately, which was executed by fractionation and direct infusion MS. Furthermore, since the DAD response varies with the molecular structure of the chromophore, either a separate external calibration with a specific standard is required for each analyte or response factors must be determined experimentally for the different compounds, as was done by Cui et al*.* [10]. Another approach is the indirect analysis *via* the analysis of fatty acid methyl esters by GC-flame ionization detector (FID) after hydrolysis and derivatization [10]. Both techniques are laborious due to the additional sample preparation steps and suffer from reduced sensitivity caused by the incomplete conversion. In addition, GC-FID analysis is more prone to errors since the measured hydrolysis products, 3-hydroxy-fatty acids, may also be derived from other molecules, such as polyhydroxyalkanoate (PHA) [20]. Hence, this GC-FID approach is unsuited to quantify the glycine-glucolipid in crude cell extracts. Quantification using HPLC-CAD offers several advantages here, such as no need for derivatization, the robustness of direct analysis, and the possibility of external calibration with a single non-specific standard substance for all glycine-glucolipid congeners. Given these advantages, HPLC-CAD has also been successfully deployed in several applications for the analysis of other biosurfactants, *i.e.*, liamocins [13], HAA, and rhamnolipids [21–24], using an inverse gradient to compensate for the detector’s mobile phase dependency [25].

Accordingly, a methodology for the analysis of glycine-glucolipids in microbial samples is presented here. It allows both the identification of congeners and their quantification, even simultaneously if desired, and thus will be of great importance for the research of this interesting biomolecule and the further development of its application potential. For this purpose, on the one hand, a method based on reversed-phase HPLC hyphenated online with HRMS was developed to enable chromatographic separation and identification of glycine-glucolipid congeners from *A. borkumensis* varying in chain length and degree of unsaturation. The retention time and characteristic fragmentation in higher energy collision-induced dissociation (HCD) were used for unambiguous identification in addition to the accurate masses. Supplementary CAD was implemented to quantify the glycine-glucolipid congeners, which, compared to DAD, does not require derivatization of the non-chromophoric analytes.

## Materials and methods

### Chemicals and material

For HPLC-MS, acetonitrile (HiPerSolv Chromanorm for LC-MS, ≥99.9%) was obtained from VWR International GmbH (Darmstadt, Germany). Ammonium formate (≥99.995% trace metals basis) and 1-Oleoyl-*rac*-glycerol (1-monoolein, ≥99%) were purchased from Sigma Aldrich Chemie GmbH (Steinheim, Germany). Formic acid (99-100% p.a.) was received from Th. Geyer (Renningen, Germany). Water was purified using a Milli-Q Academic-System (18.2 MΩ cm; 0.22-µm filter) (Millipore, Molsheim, France). For HPLC-CAD, acetonitrile (Chemsolute for LC-MS, ≥99.95%) was purchased from Th. Geyer (Renningen, Germany). Formic acid (eluent additive, ROTIPURAN ≥98.0%) was purchased from Carl Roth GmbH + Co. KG (Karlsruhe, Germany).

### Cultivation conditions and sample pretreatment

#### Bacterial strain and medium

The wild-type strain *Alcanivorax borkumensis* SK2 was used for all glycine-glucolipid production cultivations. Modified ONR7a medium contained (per L) 22.79 g NaCl, 11.18 g MgCl_2_×6 H_2_O, 3.98 g Na_2_SO_4_, 1.46 g CaCl_2_×2 H_2_O, 11.92 g HEPES, 0.72 g KCl, 2.0 g NH_4_Cl, 0.46 g of NaH_2_PO_4_×2 H_2_O, 83 mg NaBr, 31 mg NaHCO_3_, 27 mg H_3_BO_3_, 24 mg SrCl_2_×6 H_2_O, 2.6 mg NaF, and 2 mL trace elements (500×). Trace elements contained (per L) 5 g FeSO_4_×7 H_2_O, 2.5 g MnSO_4_×H_2_O, 3.2 g ZnCl_2_, 0.2 g CoCl_2_×6 H_2_O, 0.36 g CuSO_4_×5 H_2_O, 0.1 g Na_2_MoO_4_×2 H_2_O, and 6.37 g Na_2_EDTA×2 H_2_O. For a medium containing 10 g/L pyruvate as a carbon source, a 238.4 g/L HEPES stock solution with a pH of 7.0 was used, resulting in a start pH of 6.8, and for 4.83 g/L n-hexadecane as a carbon source, a 238.4 g/L HEPES stock solution with a pH of 7.8 was used, resulting in a start pH of 7.5.

For plate cultures, *A. borkumensis* SK2 was plated from a cryoculture on a marine broth agar plate with 10 g/L pyruvate and incubated for 48–72 h at 30°C. For pre-cultures, the strain was first cultivated in 100-mL shake flasks with 10 g/L pyruvate at 30°C and 200 rpm (shaking diameter: 50 mm) with a filling volume of 10% for 20–24 h. The main culture with either 10 g/L pyruvate or 4.83 g/L n-hexadecane as carbon source was inoculated to a final optical density (OD_600_) of 0.1 and cultivated at 30°C at 300 rpm (shaking diameter: 50 mm) with a filling volume of 10%.

#### Cell dry weight

For the cell dry weight (CDW), 1 mL of the culture broth was centrifuged at 4°C and 13,300 rpm for 5 min. The pellet was washed with 1 mL of bidestilled water and centrifuged under the same conditions. The supernatant was discarded. The pellet was resuspended with 1 mL bidestilled water and transferred into an HPLC vial, which was dried for 48 h and pre-weighed. The CDW was weighed after drying the sample at 65°C for 48 h.

#### Biosurfactant extraction and purification

For the biosurfactant extraction, 800 µL of the culture broth was taken, and the pH value was adjusted to pH 3.0 with 1 M HCl. The samples were mixed with 800 µL ethyl acetate and shaken on a Vortex-Genie 2 (Scientific Industries Inc., Bohemia, USA) for 10 min at room temperature. The samples were centrifuged in a Heraeus Pico 17 centrifuge (Thermo Scientific, Waltham, USA) at 17,000 × *g* for 2 min. The upper phase was transferred into a 10-mL conical tube. The extraction was repeated twice. The tubes with the organic phase were evaporated in a Scan Speed 40 speed vac (Scanspeed, Lynge, Denmark) at 800 min^−1^, 20°C, and 20 mbar for 3 h. 150 µL chloroform was added to the tube to dissolve the evaporated samples. A CHROMABOND SiOH silica gel column (200 mg/3 mL, 55 µm) (Machery-Nagel GmbH & Co. KG, Düren, Germany) was conditioned with eight column-bed volumes chloroform (2.4 mL). Then, the sample was transferred to the column and washed with 2.4 mL chloroform. The biosurfactants were eluted into a new conical tube with 13.3 column volumes (4 mL) of acetone/isopropanol (9+1, *v/v*). The eluate consisting of biosurfactants and acetone/isopropanol was evaporated under the same conditions as during the extraction. The evaporated samples were vortexed with 100 µL acetone/isopropanol (9+1, *v/v*), filtered with a 0.22-μm regenerated cellulose membrane syringe filter (Phenomenex, Torrance, USA), and measured by HPLC-MS or HPLC-CAD.

### Identification by HPLC-MS

Method development and analysis of the lipid extracts using HPLC-MS were carried out using an Ultimate 3000 UHPLC system (Thermo Scientific, Dreieich, Germany) composed of the WPS-3000 autosampler module, the SRD-3600 Degasser, the TCC-3000SD column oven, and the WPS-3000 dual gradient pump module. The HPLC system was hyphenated either to a Q Exactive Plus Hybrid Quadrupol-Orbitrap mass spectrometer (Thermo Fisher Scientific, Waltham, MA, USA) equipped with a heated electrospray ionization source (HESI-II, Thermo Scientific, Bremen, Germany) or a Corona Veo Charged-Aerosol Detector (Thermo Scientific, Dreieich, Germany) or both devices simultaneously *via* a split using a T-piece. Xcalibur 4.1 and the Dionex Chromatography MS Link plugin (Thermo Scientific) were used for HPLC-MS and HPLC-CAD instrument control. Data processing was done in Xcalibur and OriginPro 2021 (OriginLab Corporation, Northhampton, MA, USA). Samples were used for method development due to the lack of standard compounds. The use of biological samples for method development is described in detail in the “Chromatography and identification based on accurate mass” section.

Chromatographic separation using reversed-phase HPLC was performed on a Nucleodur C18 Gravity column (150×2 mm, 3 µm particle size; Macherey-Nagel, Düren, Germany). If not otherwise described, the column oven temperature was set to 60°C. Two different mobile phase conditions were evaluated: system 1 consisted of 0.2% formic acid in water as aqueous eluent (solvent A) plus 0.2% formic acid in acetonitrile as organic eluent (solvent B), and system 2 consisted of 30 mM ammonium formate buffer at pH 3 (solvent A) and acetonitrile containing 0.2% formic acid (solvent B). The following mobile phase gradient was used with system 1 for the final method: Starting with 76%B, the mobile phase composition remained constant for 0.5 min. Then, the proportion of B was linearly increased to 100% B within 36 min and held constant at 100% B for another 5 min. For re-equilibration, the gradient was changed to starting conditions within 0.5 min and kept constant for 3 min. The total runtime was 45 min. The flow rate was set to 0.31 mL/min. An injection volume of 1 µL was applied.

HRMS providing accurate mass in full scan mass mode and data-dependent fragmentation experiments was used for identification. Mass spectrometric detection was conducted with the Q Exactive Plus in combination with a HESI-II probe in positive and negative electrospray ionization modes with the following settings: capillary temperature 300°C, sheath gas flow rate 35 arbitrary units (AU), auxiliary gas flow rate 10 AU, sweep gas flow rate 1 AU, spray voltage +3.5 kV. Data were acquired in the mass range of 200–2000 *m*/*z*. In full scan mode, the mass spectrometer was operated with a resolution of 280,000 (FWHM, at *m*/*z* 200). S-lens RF level was set to 50, AGC target to 1e6, and the maximum injection time to 100 ms. Congeners were detected as deprotonated molecular ions in the negative ionization mode, and as ammonium and sodium adducts in the positive ionization mode. Structures were confirmed by data-dependent MS/MS experiments performed in a higher energy collisional dissociation (HCD) cell with a normalized collision energy (NCE) of 18 eV in negative mode or 30 eV in positive mode (based on *m*/*z* 500), unless otherwise stated. The five most intense ions or ions given in the inclusion list (with a mass deviation of 10 ppm) were selected with the quadrupole isolation width set to 1 Da. The fragmented ions as well as their isotopes were then excluded from reselection for 5 s. MS/MS spectra were acquired at a resolution of 17,500 (FWHM, at *m*/*z* 200). The AGC target was set to 1e5, and the maximum injection time to 50 ms. The minimum AGC target was 5e3, and the intensity threshold was 1e5.

### Quantification by HPLC-CAD

Biosurfactant concentration was measured using an Ultimate 3000 HPLC System with a Corona Veo Charged-Aerosol Detector (Thermo Scientific, Waltham, USA). The Nucleodur C18 Gravity column (150×3 mm, 3 µm particle size; Machery-Nagel, Düren, Germany) was used. The column oven was heated to 60°C. The injection volume was 5 µL. 0.2% formic acid (A) and acetonitrile plus 0.2% formic acid (B) were used as the mobile phase at a flow rate of 0.633 mL/min. The method lasted 46 min and included an analytical and an inverse gradient. The analytical gradient started with 24% A and 76% B for 0.5 min. Then, the ratio of B was increased to 100% within 36 min. This ratio remained constant for 5 min. Then, the ratio was changed to 24% A and 76% B within 0.5 min and was kept constant until the end of the method. The software Chromeleon (Version 7.2.10, Thermo Scientific, Waltham, USA) calculated the inverse gradient in the mode “keep solvent composition”, resulting in a flow rate of 0.633 mL/min. The inverse gradient (offset volume 778 µL) started with 100% B for 1.7 min. In the next step, the proportion of B was decreased to 76%, and A was increased to 24% within 36 min. The ratio of 24% A and 76% B was kept constant until 37.7 min. Afterward, the ratio was changed to 100% B within 0.5 min and kept constant until the end of the measurement. External calibration was performed with 1-monoolein in the 0.8 to 400 mg/L concentration range. Further details on the calibration are described in the Electronic Supplementary Material (ESM) in Fig. S34. The limits of detection (LOD) and quantification (LOQ) were established based on signal-to-noise ratios (*S*/*N*), requiring a minimum *S*/*N* of 3 for LOD and a minimum *S*/*N* of 10 for LOQ. The *S*/*N* ratio was twice the peak height divided by the signal noise.

The nitrogen pressure used for CAD was set to 57.3 psi. Detection was performed at a controlled evaporation tube temperature of 50°C with an acquisition rate of 10 Hz and a filter constant of 5 s. The power function was set to 1.00.

### Notation of glycine-glucolipid congeners

The biosurfactant from *A. borkumensis* is referred to as glycine-glucolipid. Individual congeners are named according to their structure. The term “glc” stands for the sugar unit, if any. It is followed by the total number of carbon atoms and double bonds, separated by a colon, for the tetrameric oxyacyl side chain (sum formula level). The last position marks the linked amino acid(s), usually glycine (“gly” for glycine, “ala” for alanine, and “gly_2_” for two glycine molecules connected *via* amide linkage). At the molecular congener level, the individual fatty acyl chains are designated and summed, or they are listed consecutively, separated by a slash, depending on whether the position is known (adapted from the guidelines proposed by the Lipid Maps standard initiative [26]). Aglycones that do not contain the polar head group and may be biosynthetic precursors are named accordingly, without the term “glc.”

$$\begin{array}{lc}\mathrm{Sum}\;\mathrm{formula}\;\mathrm{level}&\mathrm{glc}\text{-}42\text{:}1\text{-}\mathrm{gly}\\\mathrm{Molecular}\;\mathrm{congener}\;\mathrm{level},\;\mathrm{position}\;\mathrm{unknown}&\mathrm{glc}\text{-}10\text{:}0_{3}12\text{:}1_{1}\text{-}\mathrm{gly}\\\mathrm{Molecular}\;\mathrm{congener}\;\mathrm{level},\;\mathrm{position}\;\mathrm{known}&\mathrm{glc}\text{-}12\text{:}1/10\text{:}0/10\text{:}0/10\text{:}0\text{-}\mathrm{gly}\end{array}$$

## Results and discussion

A comprehensive and straightforward workflow has been developed to analyze glycine-glucolipids in microbial samples, allowing both the identification of congeners and their quantification. This workflow includes a method based on reversed-phase HPLC hyphenated with HRMS and a method based on reversed-phase HPLC coupled to CAD.

### Chromatography and identification based on accurate mass

**Fig. 2:**
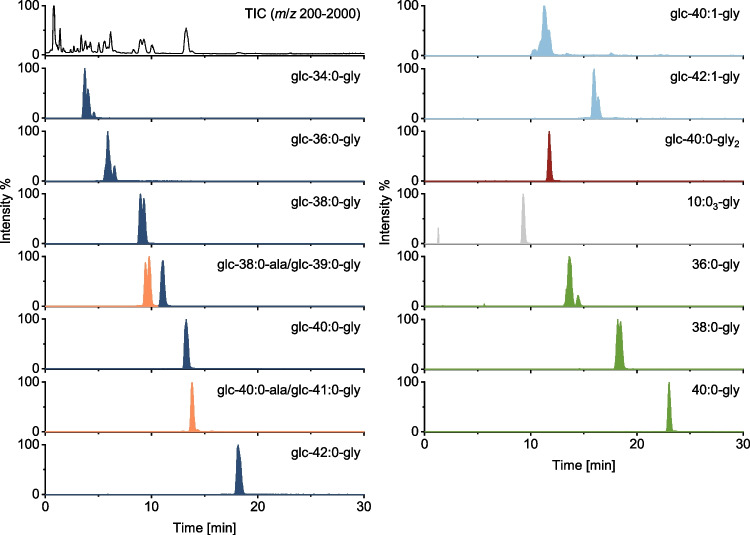
Chromatographic separation of biosurfactant congeners identified in the lipid extract from *A. borkumensis* cultivated with pyruvate. The TIC and EICs for the identified congeners (*m*/*z* 832.5064, 860.5377, 888.5690, 902.5846, 916.6003, 930.6159, 944.6316, 914.5846, 942.6159, 973.6218, 584.4168, 698.4849, 726.5162, 754.5475, mass tolerance 5 ppm) are displayed

First, a chromatographic method for species separation of glycine-glucolipid congeners and aglycones was developed, and mass spectrometric parameters were optimized. The chromatographic separation obtained with the HPLC-HRMS method is shown in Fig. 2 for the sample of *A. borkumensis* cultivated on pyruvate. The total ion chromatogram (TIC) and the extracted ion chromatograms (EICs) for 14 congeners (sum formula level, 5 ppm mass tolerance) are plotted. The negative ion mode was used for method optimization and visualization in Fig. 2 because it has higher selectivity, ionizing less matrix, and is more sensitive, with only one ion appearing at total intensity.

The method identified eight glycine-glucolipid congeners with different accurate masses, including six saturated congeners (dark blue) and two monounsaturated congeners (light blue). Three aglycone congeners (green) that do not contain a glucose moiety were also detected. Each of these groups forms its own series. The chromatographic separation is mainly based on the structure of the hydrophobic part of the molecule, the esterified hydroxyalkanoic acids, as expected when using a reversed-phase mechanism. The analytes of interest elute during the linear slope of the gradient; hence, their retention increases quite linearly with chain length (Fig. 2), which is an important additional feature for identification. Unsaturated congeners elute before the analogous saturated congeners due to the higher polarity caused by the double bond (e.g., glc-40:0-gly at 13.27 min and glc-40:1-gly at 11.25 min). The effect of the double bond may be slightly stronger or weaker depending on its localization and/or configuration (cis or trans) due to the altered spatial structure of the molecule. The aglycones that do not carry a glucose molecule as a polar head group elute significantly later than the corresponding glycine-glucolipid congeners (e.g., glc-40:0-gly at 13.27 min and 40:0-gly at 23.03 min). Furthermore, another potential biosynthetic precursor was detected (gray). This aglycone congener contains only three instead of four esterified hydroxyalkanoic acids with a total of 30 carbon atoms (*i.e.*, 10:0_3_-gly) and elutes at 9.35 min showing less retention than the tetrameric congeners. The presence of the trimeric polyhydroxyalkanoate allows speculation that as a first step in biosynthesis, the glycine is linked with a 3-hydroxy-fatty acid. Then, the other 3-hydroxy-fatty acids are iteratively attached. Finally, glucose is attached to the aglycone.

In addition, several congeners with alterations at the glycine position were identified. A congener with amide linkage to two glycine units instead of only one and several congeners alternatively linked to alanine were found. Their identification and structural characterization will be discussed in more detail in the “Verification and structural characterization based on MS/MS” section. These novel congeners show only slight changes in retention compared to the corresponding glycine-glucolipid congeners glc-40:0-gly (*i.e.*, glc-40:0-gly_2_ at 11.71 min, glc-40:0-gly at 13.27 min, and glc-40:0-ala at 13.82 min), which is consistent with expectations, since the polyhydroxyalkanoate, which is primarily responsible for the hydrophobic interaction with the column material, is identical.

Split peaks with the same accurate mass and similar full scan spectra occur for some of the identified congeners (Fig. 2). These are caused by the presence of isomers, as shown by MS/MS (“Verification and structural characterization based on MS/MS” section). These isomers either differ in the chain lengths of the bound fatty acids (chain isomers) or have the same fatty acyl composition with only the position of the individual fatty acyls varying (position isomers). The separation of all isomers, which come with more and more combination possibilities the more diverse the fatty acyl composition is, would only be possible with a massive extension of the run time, if at all, and was not targeted here.

Since some peaks show slight tailing, a different mobile phase composition containing buffer was tested. The buffer had no positive effect on either peak width or shape. The main difference was the reduced retention with buffer, which the higher elution strength can explain due to the higher salt content. Since peak widths and shapes could not be further optimized with buffer, solvent system 1 was maintained. Consequently, the congeners, or more precisely, their free carboxyl groups, appear to be present exclusively in the undissociated form, even without a buffer.

**Fig. 3:**
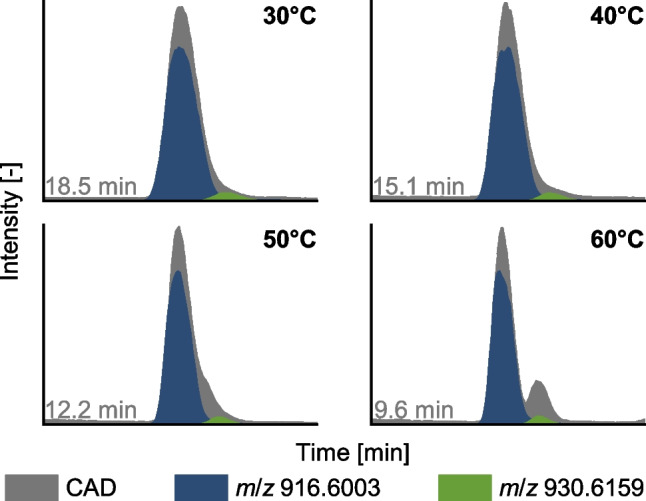
Effect of temperature on the chromatographic separation of glc-40:0-gly (*m*/*z* 916.6) and glc-40:0-ala (*m*/*z* 930.6). Since MS and CAD chromatograms were measured in different units, the scaling was chosen to fit the plot. However, the relative intensity of the EICs reflects the actual measured ratio for the sample

During method development, temperature proved to be crucial. Figure 3 shows the CAD chromatogram (gray) around the respective retention time of the main component glc-40:0-gly at temperatures of 30, 40, 50, and 60°C. Retention decreases at higher temperatures due to faster diffusion of analytes between the mobile and the stationary phases. The EICs for glc-40:0-gly (*m*/*z* 916.6003) and glc-40:0-ala (*m*/*z* 930.6159) are superimposed for each temperature. At 30°C and 40°C, the CAD chromatogram does not show the presence of two congeners because the right flank is taken for tailing, and the peak for glc-40:0-ala is completely hidden. At 50°C, a second peak can be suspected, whereas at 60°C, two peaks are visible in the CAD chromatogram. In MS, of course, both peaks can be distinguished at any temperature by looking at the EICs. However, the effect of increasing temperature on the peak widths and the chromatographic resolution of the peak pair is also clearly visible here. At 60°C, the peak area for both congeners can also be determined separately using CAD, which is essential for proper quantification (“Quantification and charged-aerosol detection” section). Higher temperatures were not tested, as 60°C is the maximum temperature recommended by the column manufacturer.

Generally, it can be concluded that a chromatographic separation of the detected congeners has been achieved, with the glycine-glucolipid congeners following a homologous series and the corresponding aglycones following their own. In the region of glc-41:0-ala and 36:0-gly, these two series overlap. However, this overlap does not prevent an unequivocal identification by MS, as they differ in exact masses. The separation is more important for non-selective CAD, which will be discussed in the “Quantification and charged-aerosol detection” section. The 15 and 12 congeners identified in the pyruvate and hexadecane culture extracts, respectively, are listed in Tables 1 and 2 together with the chemical formulas, retention times, experimentally determined and theoretical *m*/*z* values, and relative mass deviations. The absolute and relative peak areas are also provided to give a rough impression of the distribution. However, different ionization efficiencies due to different structures, matrix effects, and the changing solvent composition have not been considered. The congener distribution will be discussed in the “Quantification and charged-aerosol detection” section, together with the CAD quantification data. Additionally, the TIC and EICs supporting the identification of the congeners in the extract from hexadecane cultivation are shown in Fig. S1 in the ESM. In contrast to the pyruvate culture extract discussed above, the hexadecane culture extract additionally identified the aglycone congener 42:0-gly, which continues the homologous series of aglycones in the chromatographic separation.

**Table 1:** Identified congeners in the pyruvate culture extract of *A. borkumensis*. The congeners are denominated according to their structure. For each congener detected by HPLC-HRMS, chemical formula, retention time, experimentally determined and theoretical *m*/*z* of the deprotonated molecular ion, and the theoretical *m*/*z* value for sodium adduct (used as the precursor ion for MS/MS) are provided and supplemented by the relative mass deviation. Absolute and relative peak areas are also given to provide a rough impression of the distribution

Congener	Chemical formula	*m*/*z* calculated [M+Na]^+^	*m*/*z* calculated [M-H]^-^	Retention time (min)	*m*/*z* experimental [M-H]^-^	Deviation (ppm)	Area	Area%
glc-34:0-gly	C_42_H_75_N_1_O_15_	856.5029	832.5064	3.72, 4.03	832.5061	−0.4	4.7E+07	0.8
glc-36:0-gly	C_44_H_79_N_1_O_15_	884.5342	860.5377	5.89, 6.52	860.5373	−0.5	5.5E+08	9.9
glc-38:0-gly	C_46_H_83_N_1_O_15_	912.5655	888.5690	8.98, 9.28	888.5683	−0.8	2.0E+09	36.2
glc-38:0-ala	C_47_H_85_N_1_O_15_	926.5811	902.5846	9.45, 9.78	902.5844	−0.3	4.3E+07	0.8
glc-39:0-gly	C_47_H_85_N_1_O_15_	926.5811	902.5846	11.08	902.5849	0.3	2.9E+07	0.5
glc-40:0-gly	C_48_H_87_N_1_O_15_	940.5968	916.6003	13.27	916.5997	−0.6	2.4E+09	43.3
glc-40:0-ala	C_49_H_89_N_1_O_15_	954.6124	930.6159	13.83	930.6158	−0.2	1.5E+08	2.7
glc-42:0-gly	C_50_H_91_N_1_O_15_	968.6281	944.6316	18.15	944.6313	−0.3	9.6E+07	1.7
glc-40:1-gly	C_48_H_85_N_1_O_15_	938.5811	914.5846	11.25	914.5850	0.4	2.4E+07	0.4
glc-42:1-gly	C_50_H_89_N_1_O_15_	966.6124	942.6159	15.94, 16.34	942.6165	0.6	2.0E+07	0.4
glc-40:0-gly_2_	C_50_H_90_N_2_O_16_	997.6183	973.6218	11.74	973.6217	−0.1	1.6E+07	0.3
10:0_3_-gly	C_32_H_59_N_1_O_8_	608.4133	584.4168	9.31	584.4162	−1.0	3.0E+07	0.5
36:0-gly	C_38_H_69_N_1_O_10_	722.4814	698.4849	13.62	698.4847	−0.2	1.7E+07	0.3
38:0-gly	C_40_H_73_N_1_O_10_	750.5127	726.5162	18.20, 18.46	726.5163	0.2	6.4E+07	1.1
40:0-gly	C_42_H_77_N_1_O_10_	778.5440	754.5475	23.03	754.5477	0.3	5.0E+07	0.9

**Table 2:** Identified congeners in the lipid extract from hexadecane cultivation of *A. borkumensis*. The congeners are denominated according to their structure. For each congener detected by HPLC-HRMS, chemical formula, retention time, experimentally determined and theoretical *m*/*z* of the deprotonated molecular ion, and the theoretical *m*/*z* value for sodium adduct (used as the precursor ion for MS/MS) are provided and supplemented by the relative mass deviation. Absolute and relative peak areas are also given to provide a rough impression of the distribution

Congener	Chemical formula	*m*/*z* calculated [M+Na]^+^	*m*/*z* calculated [M-H]^-^	Retention time (min)	*m*/*z* experimental [M-H]^-^	Deviation (ppm)	Area	Area%
glc-36:0-gly	C_44_H_79_N_1_O_15_	884.5342	860.5377	5.94, 6.57	860.5376	−0.1	2.31E+07	0.5
glc-38:0-gly	C_46_H_83_N_1_O_15_	912.5655	888.5690	9.02, 9.32	888.5687	−0.3	1.98E+08	4.5
glc-39:0-gly	C_47_H_85_N_1_O_15_	926.5811	902.5846	11.09	902.5848	0.2	3.01E+07	0.7
glc-40:0-gly	C_48_H_87_N_1_O_15_	940.5968	916.6003	13.28	916.5996	−0.8	2.97E+09	67.0
glc-42:0-gly	C_50_H_91_N_1_O_15_	968.6281	944.6316	18.17	944.6311	−0.5	1.56E+08	3.5
glc-40:1-gly	C_48_H_85_N_1_O_15_	938.5811	914.5846	11.29	914.5850	0.4	3.00E+07	0.7
glc-42:1-gly	C_50_H_89_N_1_O_15_	966.6124	942.6159	15.98	942.6163	0.4	2.45E+07	0.6
glc-40:0-gly_2_	C_50_H_90_N_2_O_16_	997.6183	973.6218	11.81	973.6213	−0.5	7.03E+07	1.6
10:0_3_-gly	C_32_H_59_N_1_O_8_	608.4133	584.4168	9.34	584.4164	−0.7	3.03E+08	6.8
38:0-gly	C_40_H_73_N_1_O_10_	750.5127	726.5162	18.21	726.5161	−0.1	1.65E+07	0.4
40:0-gly	C_42_H_77_N_1_O_10_	778.5440	754.5475	23.06	754.5470	−0.6	5.88E+08	13.2
42:0-gly	C_44_H_81_N_1_O_10_	806.5753	782.5788	27.67	782.5791	0.4	1.20E+07	0.3

### Verification and structural characterization based on MS/MS

Of the identified congeners, several have not been previously described. Glc-40:0-gly, glc-38:0-gly, and glc-36:0-gly have been previously reported, including MS/MS data supporting the identification and revealing fatty acyl composition and position [9, 10]. All others are novel structures to the best of our knowledge. Therefore, the identification was subsequently corroborated by MS/MS, and the identified congeners were further characterized in terms of fatty acyl composition and position using these data. Fragmentation experiments were performed in data-dependent acquisition mode with an inclusion list to ensure that minor components were captured.

**Fig. 4:**
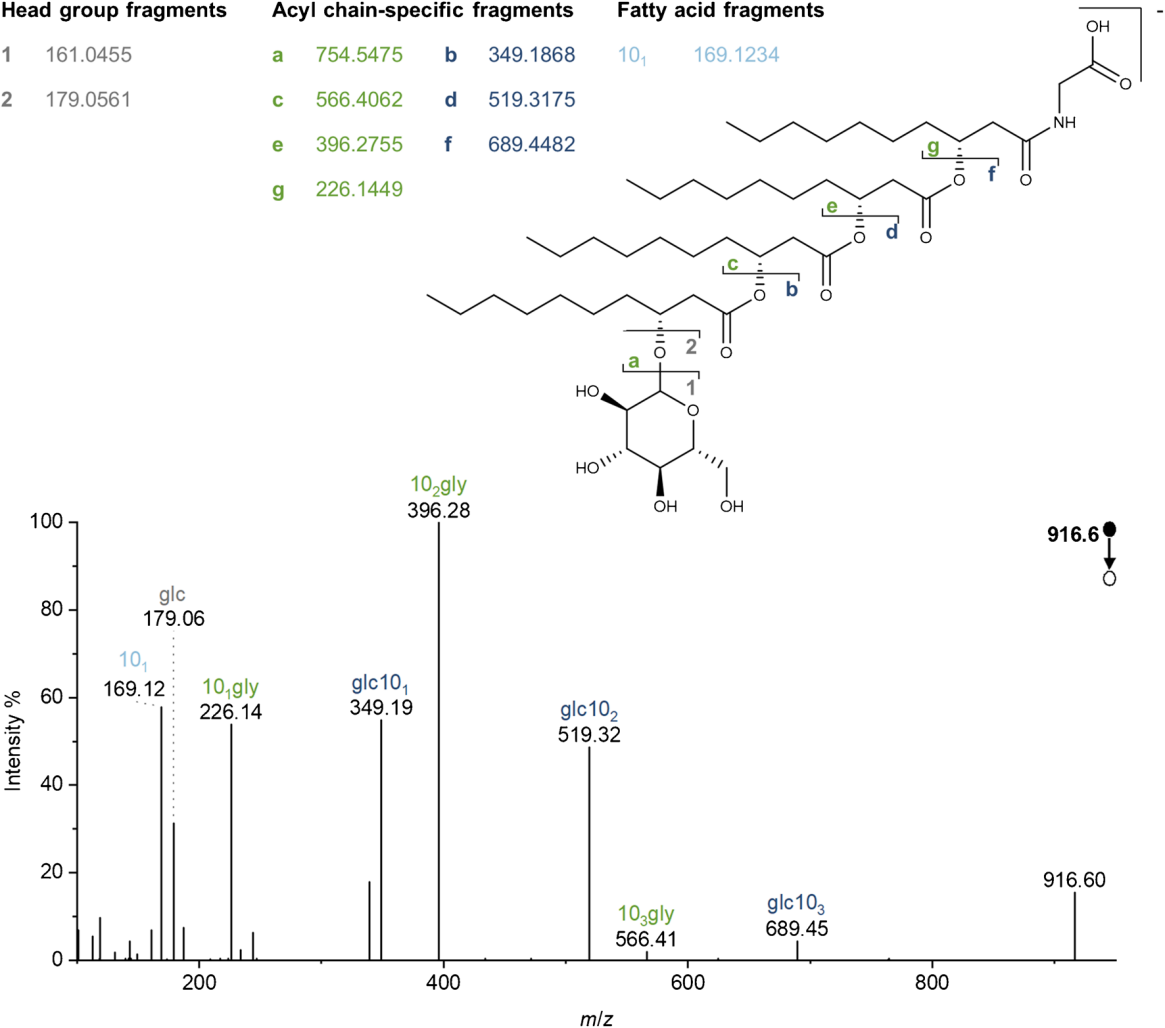
Glycine-glucolipid fragmentation in negative ion mode at the example of glc-40:0-gly eluting at a retention time of 13.26 min. The MS/MS spectrum for *m*/*z* 916.6 ([M-H]^-^) from the pyruvate culture extract is displayed. For a discussion of the observed fragments, see the text

**Fig. 5:**
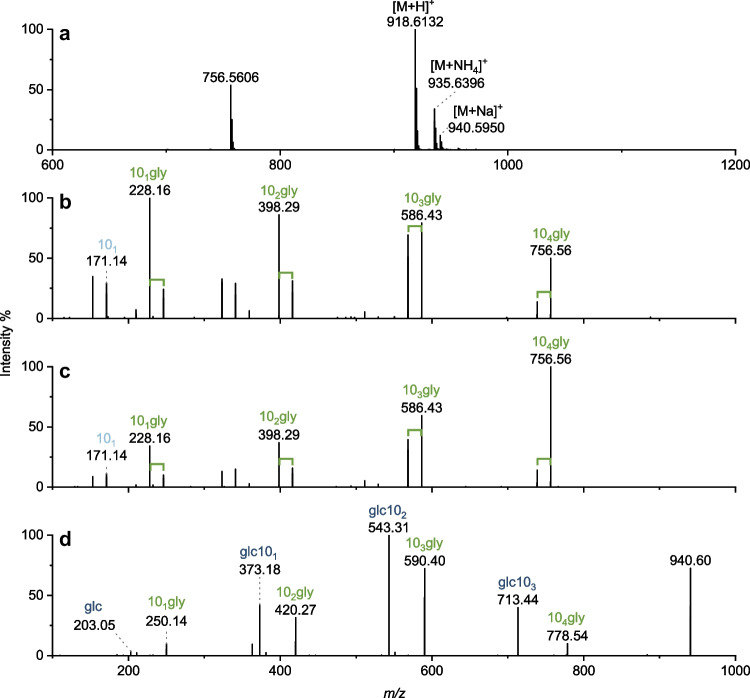
Glycine-glucolipid ionization and fragmentation in positive ion mode at the example of glc-40:0-gly. The full scan spectrum **a** and MS/MS spectra **b–d** for different precursor ions from the pyruvate culture extract are displayed. The full scan spectrum was recorded in the *m*/*z* 200–2000 mass range at 13.27 min. Fragmentation experiments were performed with HCD for (a) *m*/*z* 918.6 ([M+H]^+^) at 13.28 min and NCE 15, (b) *m*/*z* 935.6 ([M+NH_4_]^+^) at 13.31 min and NCE 15, and (c) *m*/*z* 940.6 ([M+Na]^+^) at 13.31 min and NCE 30. For a discussion of the observed fragments, see the text

**Fig. 6:**
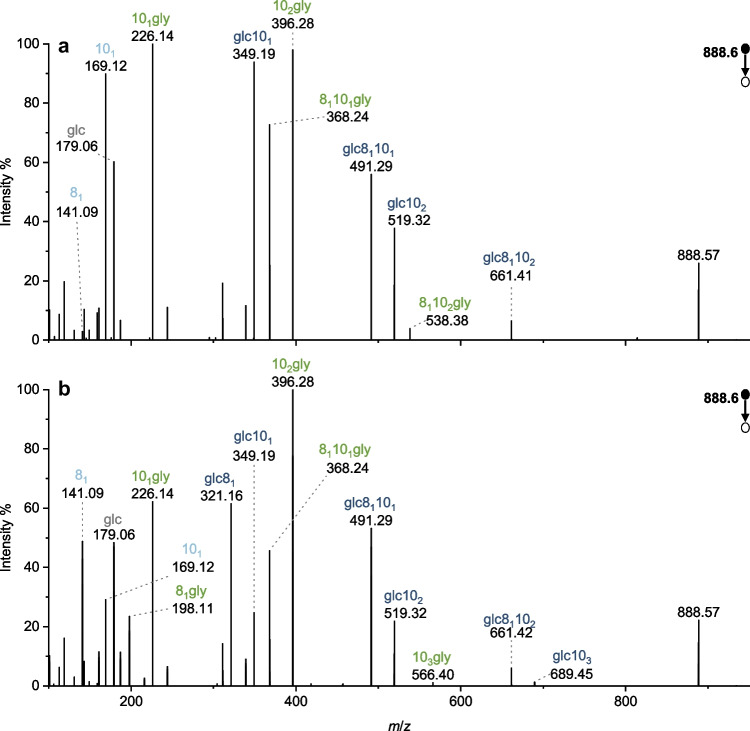
Elucidation of the isomeric structures of glc-38:0-gly underlying the two maxima of the double peak detected by HPLC-MS. MS/MS spectra from the pyruvate culture extract cultivation in negative ion mode are displayed for *m*/*z* 888.6 ([M-H]^-^) at **a** 8.90 min or **b** 9.28 min. For a discussion of the observed fragments, see the text

**Fig. 7:**
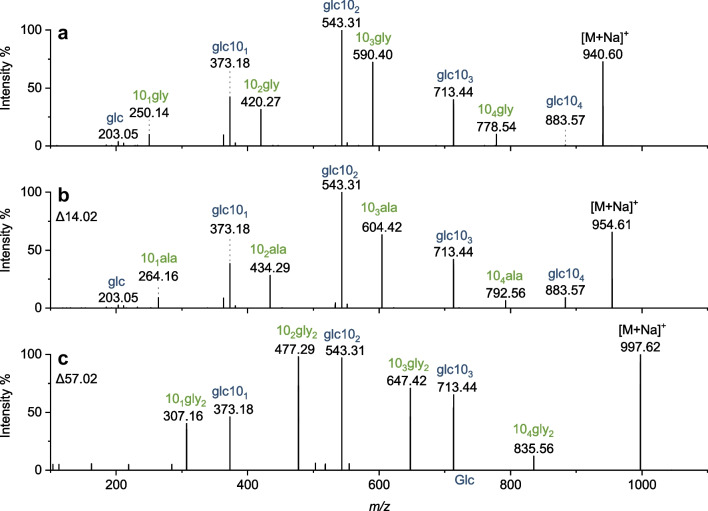
Identification of novel congeners with alterations at the amide linkage in the pyruvate culture extract. The MS/MS spectrum of the sodium adduct for **a** glc-10_4_-gly with *m*/*z* 940.6 is contrasted to those for **b** *m*/*z* 954.6 and **c** *m*/*z* 997.6. Fragmentation was performed in positive ion mode at a retention time of **a** 13.31 min, **b** 13.87, or **c** 11.75 min. For a discussion of the observed fragments, see the text

Abraham et al. [9] performed high energy-CID using a sector field tandem mass spectrometer after fast atom bombardment (FAB) in 1998. Helium was used as the collision gas to obtain the FAB-CID spectra. Here, MS/MS spectra were acquired using an Orbitrap mass spectrometer, heated electrospray ionization (HESI), and HCD. Nevertheless, the observed fragmentation pattern is similar to that described by Abraham et al*.* Therefore, the terminology of binding sites and nascent fragments was adopted (a–g), and 1 and 2 were added to denote the headgroup fragments (Fig. 4). These fragments were abbreviated according to their structure and color-coded in the MS/MS spectra displayed in Figs. 4, 5, 6, and 7 (gray for headgroup fragments, green for glycine-containing acyl chain-specific fragments, and blue for glucose-containing acyl chain-specific fragments) to facilitate understanding of the assignment.

In negative ion mode, the deprotonated molecular ion ([M-H]^-^) is the most intense and shows the characteristic fragmentation pattern. Adducts with, *e.g*., chloride were not detected in noteworthy concentrations. Figure 4 shows an MS/MS spectrum for the [M-H]^-^ of the major glycine-glucolipid of *A. borkumensis*, which is relatively straightforward to interpret due to the simple fatty acyl composition (4×10:0). *m*/*z* 179.06 and 161.05 indicate glucose without and with water loss. The exclusive occurrence of m/z 169.12 as a fatty acid fragment (after water loss) proves that only 3-hydroxy-decanoic acid was bound. Two series of characteristic fragments can be observed besides the head group fragments and the fatty acid fragments. The even-numbered series (*m*/*z* 916 → 566 → 396 → 226) contains an odd number of N-atoms derived from glycine. The series can be assigned to the fragments formed by cleavage of the glucose head group (∆162) and one to three of the attached fatty acids (∆170 *m*/*z* for C10:0). They will be referred to as glycine terminal fragments. The odd-numbered series (*m*/*z* 916 → 689 → 519 → 349) can be assigned to the fragments obtained by cleavage starting from the other side, *i.e.*, by cleavage of glycine and the terminal fatty acid (∆227) followed by cleavage of glycine and several linked fatty acids (∆170 *m*/*z* for C10:0). These fragments do not contain N-atoms and are termed glucose terminal fragments.

In positive ion mode, the fragmentation pattern is similar because the cleavage occurs at the same binding sites. Depending on which ions or adducts are observed, the corresponding *m*/*z* will vary. Figure 5 shows the MS spectrum (full scan mode), as well as the MS/MS spectra for the protonated molecular ion [M+H]^+^ (*m*/*z* 918.6), the ammonium adduct (*m*/*z* 935.6), and the sodium adduct (*m*/*z* 940.6) for the main congener glc-40:0-gly. The [M+H]^+^ gives the most intense signal in the full scan, followed by the ammonium adduct with less than 50% signal intensity. The sodium adduct gives the least intense signal. An in-source fragment resulting from the neutral loss of glucose can also be seen. This in-source fragment corresponds to the [M+H]^+^ of the corresponding aglycone congener and demonstrates the importance of chromatographic separation before fragmentation.

HCD of the [M+H]^+^ and the ammonium adduct in positive ion mode results in similar MS/MS spectra showing identical fragments with only changes in intensity. These fragments correspond to the glycine terminal fragments from negative ion mode. Here, they all appear as protonated fragments, meaning that in the case of the ammonium adduct as the precursor ion, NH_4_^+^ has been directly cleaved with the glucose moiety. The MS/MS spectrum of the sodium adduct provides more information. Analogous to the [M-H]^-^ fragmentation, glucose and glycine terminal fragments occur. All glycine terminal fragments are shifted by 23 Da compared to the other MS/MS spectra, indicating that the fragments are still present as sodium adducts. The glucose terminal fragments also each contain a sodium ion. The precursor ion was detected only in the spectrum of the sodium adduct. For the fragmentation of the others, the collision energy of the HCD should be adjusted. However, since the sodium adduct provided the most information, it was used for further characterization. The NCE was retained at 30, as it gave the best results.

When comparing the spectra from the negative and positive ion modes, both have their advantages. The spectrum from the negative ion mode shows the highest sensitivity, probably due to the high proportion of the deprotonated molecular ion species and the generally higher selectivity of the negative ion mode. Besides, the glucose and fatty acid fragments facilitate identification as a congener of this lipid class and rapid assignment of the fatty acyl composition. Fatty acid fragments are not visible in the spectrum of the sodium adduct in the positive ion mode. In contrast, the spectrum of the sodium adduct shows larger fragments, such as the fragment for 10_4_gly, and higher intensities for the fragments 10_3_gly and glc10_3_. This information complements well the information from the negative mode. Using the spectra from both modes, the identified congeners were characterized in more detail.

It was then necessary to clarify whether the split peaks of the same *m*/*z* mentioned in the “Chromatography and identification based on accurate mass” section were due to the glycine-glucolipid congener or isobaric or isomeric interferences. Therefore, it was investigated how the multiple peaks of one *m*/*z* differ. Figure 6 contrasts the MS/MS spectra obtained in negative ion mode for the [M-H]^-^ of glc-38:0-gly with *m*/*z* 888.6. The MS/MS spectra both show the expected fragments resulting from the specific fragmentation pattern confirming their identification as glycine-glucolipids. Many of the detected fragments are common to both spectra. The 3-hydroxy-octanoic acid and 3-hydroxy-decanoic acid fragments are detected in both spectra, while no other fatty acid fragments appear. The acyl chain-specific fragments do not indicate the presence of other fatty acids either. Thus, the congeners underlying the two peaks do not appear to differ in fatty acyl composition. However, the fragments for glc8_1_ and glc10_3_ as well as the fragments 8_1_gly and 10_3_gly can only be observed in the lower spectrum acquired at 9.28 min. The fragment 8_1_10_2_gly, on the other hand, only appears in the upper spectrum obtained at 8.90 min. This selective appearance of these fragments suggests that the fatty acyl positions are different at different retention times. The upper spectrum suggests that the 3-hydroxy-octanoic acid is in one of the two middle positions of the polyester tail. The lower spectrum suggests that it is located at either end of the tetrameric structure.

As briefly mentioned in the “Chromatography and identification based on accurate mass” section, congeners with modifications at the glycine end were identified. At 13.80 min, a signal with *m*/*z* 930.6158 was detected in negative ion mode (Fig. 2). From accurate mass alone, this *m*/*z* would match the exact mass of the deprotonated molecular ion of glc-41:0-gly (*m*/*z* 930.6159, mass deviation 0.2 ppm, Table 1). However, the retention time is inconsistent with that predicted for glc-41:0-gly based on the homologous series. The MS/MS spectra in both modes (Fig. 7b and ESM, Fig. S2) show fragments known from the other glycine-glucolipids. They show the glucose terminal fragments analogous to the fragmentation of glc-40:0-gly. However, the precursor ion and the glycine terminal fragments are shifted by about 14.02 Da, corresponding to CH_2_. This shift would be consistent with 3-hydroxy-undecanoic acid proximal to glycine as well as 3-hydroxy-decanoic acid and alanine attached instead of glycine. However, in negative mode, no fatty acid fragment was detected to prove the presence of 3-hydroxy-undecanoic acid (ESM, Fig. S2). Also, the fragment for glc10_4_ with *m*/*z* 883.57 in the MS/MS spectrum from the positive ion mode proves that only 3-hydroxy-decanoic acid is present, thus confirming the alteration at the amide linkage (Fig. 7b). It was hypothesized that the binding of alanine instead of glycine is responsible for the increase in the mass of CH_2_. An analogous shift was observed for the *m*/*z *902.5846, corresponding to glc-39:0-gly and glc-38:0-ala, at 9.41 min and 9.77 min (ESM, Table S1). Thus, alanine modification is assumed for those signals. For the signal at 11.12 min, on the other hand, glc-39:0-gly is confirmed, since, among others, the 3-hydroxy-nonanoic acid fragment is also detected (ESM, Table S1). Furthermore, the retention time fits into the homologous series.

Another congener was detected at 11.74 min with MS/MS spectra again showing the glucose terminal fragments known from the fragmentation of glc-40:0-gly. This time, the precursor ion and the glycine terminal fragments are shifted by about 57.02 Da towards higher *m*/*z* (Fig. 7c and ESM, Fig. S3). This fragmentation would be consistent with the expected fragmentation for an analogous congener with two glycine units attached. The accurate masses obtained from the full scan (*m*/*z* 973.6217 or 973.6213) also agree with the molecular formula of the postulated structure with an exact mass of 973.6218 with a mass deviation well below 1 ppm for both samples (Tables 1 and 2). Further analysis should be performed in the future to confirm these novel structures, *e.g.*, by NMR analysis of the isolated congener. Also, amino acid analysis by GC-FID after hydrolysis of the isolated congener could confirm alanine. However, these investigations were not performed in this study because these congeners were less relevant for biotechnological optimization regarding yield and the quantification of the total biosurfactant content due to their very low content.

The fragmentation of the aglycones is similar, but only glycine terminal fragments can be detected. The MS/MS spectrum for the aglycone 42:0-gly is shown in the ESM in Fig. S4 in negative ion mode. In contrast to the other spectra (Figs. 4 and 6), the fatty acid fragments do not exhibit any water loss. Therefore, their *m*/*z* are shifted by ∆18 towards higher *m*/*z* values (theoretical *m*/*z* 10_1_ = 187.1334, theoretical *m*/*z* 12_1_ = 215.1647). This difference is probably because the hydroxy group is not cleaved with the attached glucose, suggesting that the signal is derived solely from the terminal fatty acyl group. From the example shown, it can be concluded that 42:0-gly contains C10:0- and C12:0-fatty acids, both of which can be terminal or proximal to glycine.

In summary, 16 congeners of this lipid type, *i.e.*, glycine-glucolipid congeners including related structures like aglycones and congeners with glycine alterations, were detected in one or both samples of *A. borkumensis* (Tables 1 and 2). Congener identification based on accurate mass and retention time was confirmed by MS/MS results (Table S1–Table S4). In addition, the fatty acyl composition was determined by MS/MS and the respective positions were assigned where unambiguous MS/MS spectra were available (Table 3). However, assignment on fatty acyl position level was only possible for a few congeners in this example. Options to obtain MS/MS spectra for all position isomers individually, which would allow an unambiguous assignment of fatty acyl composition and position, could be either the preparative or analytical separation of the individual structures at a massive extent of time, if at all possible, or the biosynthesis of samples that do not yield a mixture of different fatty acyl position isomers.

**Table 3:** Detailed characterization of the identified congeners from pyruvate and hexadecane cultivation of *A. borkumensis* based on MS/MS. Characterization was performed for the individual retention times of the double peaks, where MS/MS could be recorded. The conclusions from negative and positive ion modes regarding the composition and position of the fatty acyl groups are summarized

Congener	Pyruvate cultivation, negative ion mode			Pyruvate cultivation, positive ion mode			Hexadecane cultivation, negative ion mode			Hexadecane cultivation, positive ion mode		
	Rt (min)	Chain level	Position level	Rt (min)	Chain level	Position level	Rt (min)	Chain level	Position level	Rt (min)	Chain level	Position level
glc-34:0-gly	3.72	8_3_10_1_	Non-specific	3.75	8_3_10_1_	Non-specific						
	4.03	6_1_8_1_10_2_	6/10/8/10, 6/8/10/10	4.05	No MS/MS	No MS/MS						
glc-36:0-gly	5.89	8_2_10_2_	Non-specific	5.87	8_2_10_2_	Non-specific	5.94	8_2_10_2_	Non-specific	5.90	No MS/MS	No MS/MS
	6.52	6_1_10_3_	6/10/10/10	6.52	6_1_10_3_	6/10/10/10	6.57	6_1_10_3_	6/10/10/10	6.50	No MS/MS	No MS/MS
glc-38:0-gly	8.98	8_1_10_3_	10/8/10/10, 10/10/8/10	8.95	8_1_10_3_	10/8/10/10, 10/10/8/10	9.02	8_1_10_3_	10/8/10/10, 10/10/8/10	8.96	8_1_10_3_	10/8/10/10, 10/10/8/10
	9.28	8_1_10_3_	8/10/10/10, 10/10/10/8	9.29	8_1_10_3_	8/10/10/10, 10/10/10/8	9.32	8_1_10_3_	8/10/10/10, 10/10/10/8	9.27	8_1_10_3_	8/10/10/10, 10/10/10/8
glc-38:0-ala	9.45	8_1_10_3_	10/8/10/10, 10/10/8/10,	9.49	8_1_10_3_	10/8/10/10, 10/10/8/10						
	9.78	8_1_10_3_	8/10/10/10, 10/10/10/8	9.88	8_1_10_3_	8/10/10/10, 10/10/10/8						
glc-39:0-gly	11.08	9_1_10_3_	Non-specific	11.07	9_1_10_3_	Non-specific	11.09	9_1_10_3_	Non-specific	11.03	9_1_10_3_	Non-specific
glc-40:0-gly	13.27	10_4_	10/10/10/10	13.29	10_4_	10/10/10/10	13.28	10_4_	10/10/10/10	13.29	10_4_	10/10/10/10
glc-40:0-ala	13.83	10_4_	10/10/10/10	13.83	10_4_	10/10/10/10						
glc-42:0-gly	18.15	10_3_12_1_	10/10/10/12, 12/10/10/10	18.13	10_3_12_1_	10/10/10/12, 12/10/10/10	18.17	10_3_12_1_	10/10/10/12, 12/10/10/10	18.14	10_3_12_1_	10/10/10/12, 12/10/10/10
glc-40:1-gly	11.25	10_3_10:1_1_	Non-specific, glc10_1_	11.29	No MS/MS	No MS/MS	11.29	10_3_10:1_1_	Non-specific, glc10_1_	11.20	No MS/MS	No MS/MS
glc-42:1-gly	15.94	10_3_12:1_1_	Non-specific, glc10_1_	15.92	10_3_12:1_1_	Non-specific, glc10_1_	15.98	10_3_12:1_1_	Non-specific, glc10_1_	15.93	10_3_12:1_1_	Non-specific, glc10_1_
	16.34	10_3_12:1_1_	12:1/10/10/10	16.32	No MS/MS	No MS/MS						
glc-40:0-gly_2_	11.74	10_4_	10/10/10/10	11.75	10_4_	10/10/10/10	11.81	10_4_	10/10/10/10	11.75	10_4_	10/10/10/10
30:0-gly	9.31	10_3_	10/10/10	9.24	10_3_	10/10/10	9.34	10_3_	10/10/10	9.27	10_3_	10/10/10
	13.62	8_2_10_2_	Non-specific	13.61	8_2_10_2_	Non-specific						
38:0-gly	18.20	8_1_10_3_	Non-specific, 10_1_gly	18.18	8_1_10_3_	Non-specific, 10_1_gly	18.21	8_1_10_3_	Non-specific, 10_1_gly	18.15	8_1_10_3_	Non-specific, 10_1_gly
	18.46	8_1_10_3_	Non-specific, 8_1_gly	18.43	No MS/MS	No MS/MS						
40:0-gly	23.03	10_4_	10/10/10/10	23.02	10_4_	10/10/10/10	23.06	10_4_	10/10/10/10	23.04	10_4_	10/10/10/10
42:0-gly							27.67	10_3_12_1_	Non-specific	27.69	10_3_12_1_	Non-specific

*For saturated congeners, the number of double bonds has been omitted in the shorthand notation at the molecular species level for better readability

### Quantification and charged-aerosol detection

**Fig. 8:**
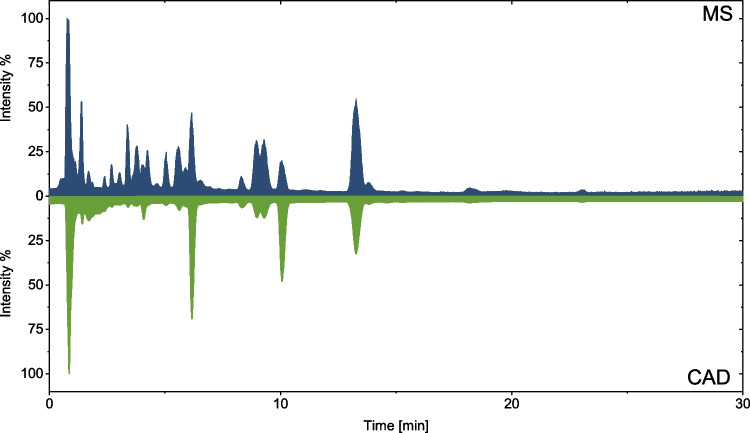
Comparison of MS and CAD measurements for signal assignment in CAD. The TIC from negative ion mode from MS and the CAD chromatogram are contrasted

Complementarily, universal CAD was implemented for quantifying glycine-glucolipid congeners and aglycones, as authentic standards were not available for MS quantification. In contrast to DAD, CAD does not require derivatization of the non-chromophore analytes and offers better sensitivity and dynamic range than ELSD [27]. The CAD signals could be assigned by matching retention times in reversed-phase HPLC. As expected for reversed-phase HPLC, the retention times for the developed method were stable and in good agreement for MS and CAD. Fig. 8 compares the TIC from the negative ion mode and the CAD chromatogram for simultaneous measurement using a T-piece.

The nearly uniform detector response for non-volatile analytes, with no substantial alteration from changing spectral or physicochemical properties of the analyte, allowed quantification by external calibration with a structurally related standard compound, provided that the mobile phase composition was consistent throughout the run [25, 27, 28]. In this case, 1-monoolein was used as external standard. To ensure consistency of the mobile phase composition, an inverse gradient was used for mobile phase compensation for quantitative measurements. Therefore, all congeners of that lipid type can be quantified with only one external standard curve. A calibration series was measured with 1-monoolein between 0.8 and 400 mg/L (3.9–2000 ng on the column) (ESM, Fig. S34). After ethyl acetate extraction and subsequent SPE purification, all samples were concentrated 8-fold. Thus, the lowest and highest measured concentration of glycine-glucolipids was in the range of the calibration series. Furthermore, LOD and LOQ were determined, with 3-fold *S*/*N* for LOD and 10-fold *S*/*N* for LOQ. LOD was 0.15 mg/L, and LOQ was 0.5 mg/L, which is within the range specified by Thermo Fisher for the CAD for the detection limit. Thus, the method is valid for quantifying the congeners visible in the CAD of the natural glucolipid mixture of *A. borkumensis* SK2. The sensitivity of the presented HPLC-CAD method is at least one order of magnitude lower than that of methods with mass spectrometric detection [21]. For this reason, not all congeners found using MS could be quantified with the HPLC-CAD method. However, the quantification of minor components was not the aim of the study but to enable quantification of the glycolipid of *A. borkumensis* without derivatization of the sample. Congeners only detectable by MS and not by CAD are present only in trace concentrations and, therefore, are less relevant for optimizing the fermentation process.

**Fig. 9:**
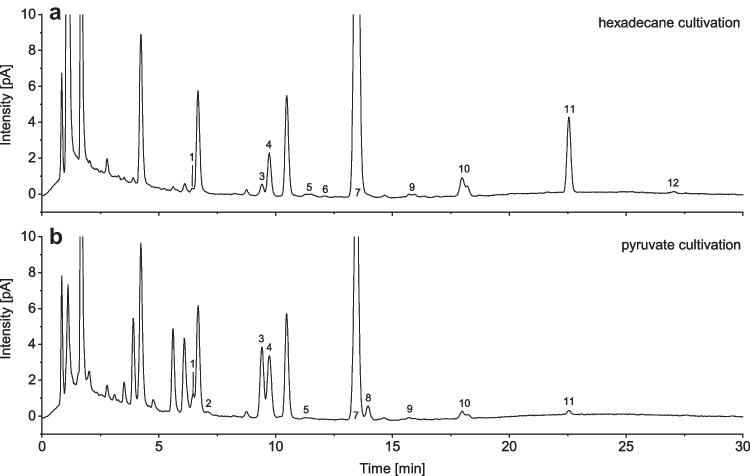
Inverse gradient CAD chromatograms for **a** hexadecane cultivation and **b** pyruvate cultivation. 1, glc-36:0-gly; 2, glc-36:0-gly; 3, glc-38:0-gly; 4, glc-38:0-gly+10:0_3_-gly; 5, glc-39:0-gly+glc-40:1-gly; 6, glc-40:0-gly_2_; 7, glc-40:0-gly; 8, glc-40:0-ala; 9, glc-42:1-gly; 10, glc-42:0-gly+38:0-gly; 11, 40:0-gly; 12, 42:0-gly

**Fig. 10:**
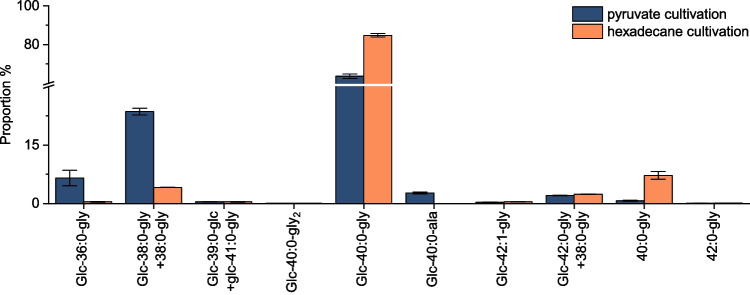
Congener distribution determined by HPLC-CAD compared for pyruvate and hexadecane cultivation (*n* = 3)

After both cultures were in the stationary growth phase, the culture broth was acidified and extracted three times with ethyl acetate. Afterward, the extract was purified *via* SPE and was ready for HPLC-CAD measurement. Fig. 9 shows the HPLC-CAD chromatograms and signal assignment for the cultivation with pyruvate and hexadecane as carbon sources. Twelve different signals could be assigned in the CAD chromatogram, but the following congeners glc-38:0-gly+10:0_3_-gly, glc-39:0-gly+glc-40:1-gly, and glc-42:0-gly+38:0-gly coelute (Fig. 9) and are therefore reported as sum parameters (Fig. 10). The coelution does not interfere with quantification in the biotechnological process because glucolipids are always produced as a natural mixture of congeners. Ultimately, a sum concentration of all congeners is usually presented in the literature [29, 30]. Since only glycine-glucolipid and aglycone congeners coelute and not matrix components, the total peak area can be counted in each case for the total amount of biosurfactant produced.

Figure 10 shows the congener distribution of glucolipids, including aglycones determined from the CAD peak areas, using second-order polynomial regression of the external calibration. These results should be more accurate than determining proportions based on MS peak areas because the ionization efficiency is not a factor here; due to the inverse gradient, all analytes have nearly the same CAD response at the same mass concentration [27]. Differences between cultivation with pyruvate and hexadecane can be seen. The main congener for both carbon sources is glc-40:0-gly, with 64% for pyruvate and 85% for hexadecane. Furthermore, there are differences in the shorter-chain glycine-glucolipids, which are only produced in small amounts during the cultivation with hexadecane. With pyruvate, the congeners glc-36:0-gly and glc-38:0-gly+10:0_3_-gly are produced at 7% and 24%, respectively. These findings are in good agreement with those obtained by Cui et al*.* [10] using HPLC-DAD. In contrast, glycine-glucolipids and precursor molecules (aglycones) containing a 3-hydroxy-dodecanoic acid were found in this study. Interestingly, the congener glc-40:0-ala was found only when grown with pyruvate. Furthermore, no glycine-free form of the glucolipid was found in this study either [1, 9, 10]. The glycine-free form of glucolipid was first described in the literature by Passeri et al*.* [1] in 1992 and not since. One explanation for why the glycine-free form was not found in subsequent studies could be that the glucolipid was purified under harsh conditions, resulting in glycine cleavage. Thus, the glycine-free form could have been formed based on an abiotic effect. This abiotic effect could be related to the pH shift to 3 with a strong acid (H_2_SO_4_), and the glycine could have been cleaved from the glucolipid by hydrolysis, which could be the case with a longer extraction process. Unfortunately, Passeri et al*.* [1] do not provide any information on the extraction time or temperature. In our study, the extraction time was short, and the solvent from the extraction was evaporated quickly after extraction (compare the “Cultivation conditions and sample pretreatment” section).

The main difference between the cultivations is the carbon source. Pyruvate is a C_3_ molecule and water-soluble. Hexadecane is a C_16_ molecule and water-insoluble and therefore forms a second phase. Table 4 shows the performance parameters for both cultivations in which the substrate concentrations were adjusted to the same molar amount of carbon. The glucolipid titer with hexadecane as a carbon source is twice as high as pyruvate, 49 mg/L *versus* 22 mg/L. Additionally, less biomass is formed with hexadecane, resulting in a specific yield (*Y*_P/X_ = 19 mg/g) that is three times higher than with pyruvate. Biosurfactant production is a response to water-insoluble substrates found in the natural environment, allowing bacteria to access these compounds as a carbon source. *A. borkumensis* can predominantly utilize alkanes as a carbon source, producing a glycine-glucolipid associated with the cell membrane and increasing hydrophobicity of the cell surface. The higher cell surface hydrophobicity enables the cells to adhere more effectively to the alkane droplets, resulting in better uptake and, ultimately, more effective degradation of water-insoluble substances [2, 3, 10, 31–33]. That *A. borkumensis* produces more glycine-glucolipid per cell on hexadecane than with pyruvate is consistent with the results of Cui et al*.* [10]. However, the glucolipid titer with hexadecane as a carbon source was reported to be 0.4-fold lower than that with pyruvate [10]*.* Despite the same growth medium, our study could not confirm the lower glucolipid titer on hexadecane compared to pyruvate. Several reasons may be responsible for the lower glucolipid titer on hexadecane in Cui et al*.* [10]. Firstly, a different extraction method (chloroform/methanol) was used, which may not have a high extraction efficiency. A pH shift to 3.0 prior to ethyl acetate extraction seems beneficial for the biosurfactant extraction from the culture broth. The literature often describes ethyl acetate extraction with acidic pH as a simple and highly efficient biosurfactant extraction method [34, 35]. Secondly, the cultures were likely oxygen-limited, shaken at 180 rpm with a filling volume of 20%. As Schlosser et al*.* [36] described, the oxygen transfer rate may influence biosurfactant production. In addition to the lower oxygen transfer rate, the mixing of the second phase, in this case, hexadecane, is also significantly reduced. Thus, the degradation occurs more slowly and incompletely and, therefore, also affects the production of the biosurfactant. These reasons may explain why the titer of Cui et al*.* [10] differs from our study.

**Table 4:** Comparison of glycolipid titer, cell dry weight (CDW), product-to-biomass yield (*Y*_P/X_), and product-to-substrate yield (*Y*_P/S_) for the pyruvate and hexadecane cultivation with *A. borkumensis* SK2 (*n* = 3)

Cultivation	Substrate concentration (g/L)	Titer (mg/L)	CDW (g/L)	Y_P/X_ (mg/g)	Y_P/S_ (mg/Cmol)
Pyruvate	10	22 ± 4	3.7 ± 0.0	6	65
Hexadecane	4.8	49 ± 5	2.6 ± 0.3	19	144

It is necessary for the bioprocess development of *A. borkumensis* to have a detection method that can quantify glycine-glucolipids and the aglycones independently of their chemical structure, such as DAD or CAD. When combined with derivatization, DAD detection is a selective method. However, the derivatization process to obtain phenacyl esters of the glucolipid is time-consuming and requires additional manual preparation steps, limiting its throughput. It also necessitates extra chemicals, laboratory equipment, and an excess of derivatization reagents to ensure complete derivatization. Additionally, in the reversed-phase chromatographic method used in this study, some glycine-glucolipids congeners could, even more, coelute due to the phenacyl ester group reducing structural differences. The phenacyl esters of glycine-glucolipids and aglycones are more hydrophobic than the natural glucolipid, resulting in longer retention times. These reasons would necessitate a thorough re-optimization of the chromatographic method.

On the other hand, CAD is a universal method for non-volatile analytes. Inverse gradient compensation requires a more complex instrumental setup, including a second time-balanced pump. However, it eliminates the need for derivatization and adaptation of the chromatographic method. Furthermore, the uniform response of CAD enables quantification even when pure standard compounds are unavailable [21]. As a result, non-volatile compounds, such as 1-monoolein, can serve as suitable universal standards. Thus, HPLC-CAD presents an appealing alternative to HPLC-DAD detection.

## Conclusions

A method based on reversed-phase HPLC hyphenated with HRMS to enable chromatographic separation and identification of the glycine-glucolipid congeners from the marine bacterium *A. borkumensis* SK2 varying in chain length and degree of unsaturation was developed. In addition to accurate mass, retention time and characteristic fragmentation in positive and negative ion modes were used for unambiguous identification. CAD was utilized for complementary quantification, which, compared to DAD, does not require the derivatization of the non-chromophoric analytes. The methodology was then applied to investigate the biotechnological production of the glycine-glucolipid using different carbon sources, *i.e.*, pyruvate and hexadecane. Congeners that differ in chain length (C_6_–C_12_, also odd-numbered) and degree of unsaturation (0–1 double bonds) of the bound 3-hydroxy-alkanoic acids were identified, some of which, to our knowledge, have not been previously reported. In addition, other congeners were discovered by MS/MS experiments postulated to be altered at the amide bond, *e.g.*, the substitution of glycine with alanine or two glycine units. In the future, the new structures should be confirmed by NMR analysis after purification, which would likely require numerous injections to collect sufficient amounts of the minor components or the development of a preparative HPLC method. Complementary quantification shows that the titer was approximately twice as high for hexadecane as for pyruvate (49 mg/L *versus* 22 mg/L). For the first time, a glycine-glucolipid titer from *A. borkumensis* could be measured directly without relying on correction factors and derivatization. The direct measurement was only possible due to the HPLC setup with an inverse gradient, charged-aerosol detection, and hyphenated HRMS to identify the different congener retention times. Furthermore, the specific yield was also higher when grown with hexadecane at 19 mg/g cell dry weight and 144 mg/Cmol substrate. In both cases, the main congener produced was glc-40:0-gly, with a proportion of 64% for pyruvate and 85% for hexadecane. When cultivated with pyruvate, 3-hydroxy-octanoic acid, in addition to 3-hydroxy-decanoic acid, was incorporated to a greater extent compared to cultivation with hexadecane; with pyruvate, the congeners glc-36:0-gly and glc-38:0-gly+10:0_3_-gly are formed at 7% and 24%, respectively. Furthermore, the occurrence of possible biosynthetic precursors of the glycine-glucolipid in the lipid extracts was analyzed. No evidence for a glycine-free form of the glucolipid was found, but 10:0_3_-gly was detected. The detection of a trimeric aglycone congener and several tetrameric aglycones could be an important clue for the future elucidation of the biosynthetic pathway.

### Supplementary Information

Below is the link to the electronic supplementary material.

Supplementary file1 (PDF 889 KB)
