# Supplementary material for: Identification and quantification of biosurfactants produced by the marine bacterium Alcanivorax borkumensis by hyphenated techniques
Source: Anal Bioanal Chem. 2023 Oct 11;415(29-30):7067–84. doi: 10.1007/s00216-023-04972-5 (PMC10684412; doi:10.1007/s00216-023-04972-5)
Supplement: Supplementary file 1 — Supplementary file1 (PDF 889 KB) [file 216_2023_4972_MOESM1_ESM.pdf]

Analytical and Bioanalytical Chemistry
https://doi.org/10.1007/s00216-023-04972-5

Electronic Supplementary Material

Identification and quantification of biosurfactants produced by the marine bacterium Alcanivorax borkumensis by hyphenated techniques

Anna Lipphardt[1], Tobias Karmainski[2], Lars M. Blank[2], Heiko Hayen[1], Till Tiso[2]
[1]Institute of Inorganic and Analytical Chemistry, University of Münster, Münster, Germany
[2]Institute of Applied Microbiology, RWTH Aachen University, Aachen, Germany
Email corresponding author: till.tiso@rwth-aachen.de

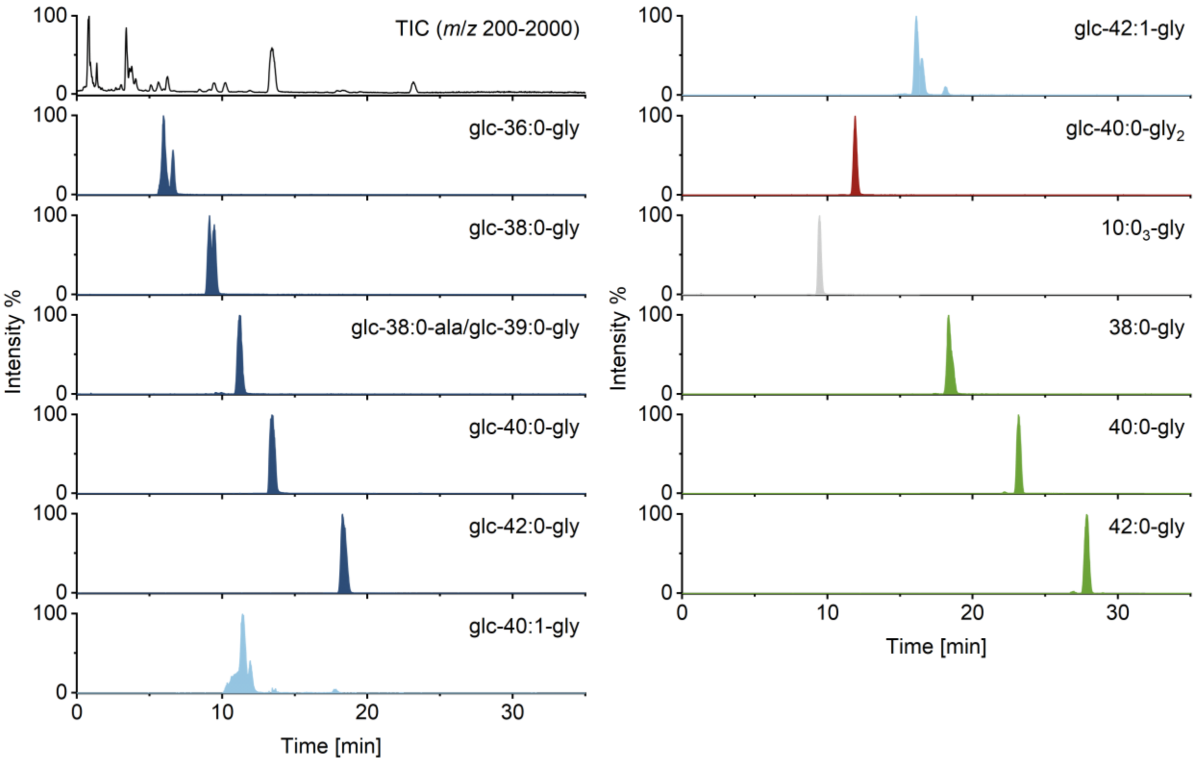

Fig. S1 Chromatographic separation of species identified in lipid extract from *A. borkumensis* cultivated with hexadecane. The TIC and EICs for the identified species (*m/z* 860.5377, 888.5690, 902.5846, 916.6003, 944.6316, 914.5846, 942.6159, 973.6218, 584.4168, 726.5162, 754.5475, 782.5788 (mass tolerance 5 ppm) are displayed

Table S1 Detailed characterization of the identified species from pyruvate cultivation of *A. borkumensis* based on MS/MS. Characterization was performed for the individual retention times of the double peaks, where MS/MS could be recorded. The fragments detected in negative ion mode are listed, and the conclusions for the composition and position of the fatty acyl groups are summarized

**Species**	**Rt /min**	**[M-H]$^-$**	**FS**	**1 Glc**	**2 Glc ($-H_2O$)**	**b $GlcFA_1$**	**c FA_3Gly**	**d $GlcFA_2$**	**e FA_2Gly**	**f $GlcFA_3$**	**g FA_1Gly**	**Fatty acyl composition**	**Fatty acyl position**
Glc-34:0-Gly	3.72	832.51	141.09, 169.12	179.06	161.05	321.15, 349.19		463.25, 491.29	340.21, 368.24		198.11, 226.14	$8_3 10_1$	non-specific
	4.03	832.50	113.06, 141.09, 169.12	179.06		293.12		435.22, 463.25, 491.29	340.21, 368.24, 396.28	605.36	226.14	$6_1 8_1 10_2$	6/10/8/10, 6/8/10/10
Glc-36:0-Gly	5.89	860.54	141.09, 169.12	179.06	161.05	321.16, 349.19	538.38	463.25, 491.29, 519.32	340.21, 368.24, 396.28	633.39, 661.42	198.11, 226.14	$8_2 10_2$	non-specific
	6.52	860.54	113.06, 169.12	179.06	161.05	293.12, 349.19	566.41	463.25, 519.32	340.21, 368.24, 396.28	633.38	170.08, 226.14	$6_1 10_3$	6/10/10/10
Glc-38:0-Gly	8.98	888.57	141.09 169.12	179.06	161.05	349.19	538.38	491.29, 519.32	368.24, 396.28	661.41	226.14	$8_1 10_3$	10/8/10/10, 10/10/8/10
	9.28	888.57	141.09, 169.12	179.06	161.05	321.16, 349.19	566.40	491.29, 519.32	368.24, 396.28	661.42, 689.45	198.11, 226.14	$8_1 10_3$	**8/10/10/10,** 10/10/10/8
Glc-38:0-Ala	9.45	902.59	169.12	179.06	161.05	349.19	552.39	491.28, 519.32	382.26, 410.29	661.42	240.16	$8_1 10_3$	10/8/10/10, 10/10/8/10,
	9.78	902.59	141.09, 169.12	179.06	161.05	321.16, 349.19	580.42	491.29, 519.32	382.26, 410.29	661.41	212.13, 240.16	$8_1 10_3$	**8/10/10/10**, 10/10/10/8
Glc-39:0-Gly	11.08	902.58	155.11, 169.12	179.06	161.05	335.17, 349.19	552.39	505.30, 519.32	382.26, 396.28	675.43	212.13, 226.14	$9_1 10_3$	non-specific
Glc-40:0-Gly	13.27	916.60	169.12	179.06	161.05	349.19	566.41	519.32	396.28	689.45	226.14	10_4	10/10/10/10
Glc-40:0-Ala	13.83	930.62	169.12	179.06	161.05	349.19	580.42	519.32	410.29	689.45	240.16	10_4	10/10/10/10
Glc-42:0-Gly	18.15	944.63	169.12, 197.15	179.06	161.05	349.19, 377.22	566.40	519.32, 547.35	396.28, 424.31	689.45, 717.48	226.14, 254.18	$10_3 12_1$	10/10/10/12, 12/10/10/10
Glc-40:1-Gly	11.25	914.58	167.11, 169.12	179.06	161.05	349.19	564.39	491.29, 517.30, 519.32	394.26, 396.27, 422.29		224.13 226.14	$10_3 10{:}1_1$	non-specific, $Glc10_1$
Glc-42:1-Gly	15.94	942.61	169.12	179.06	161.05	349.19		519.32, 545.33	396.27, 422.29	689.44	226.14, 252.16	$10_3 12{:}1_1$	non-specific, $Glc10_1$
	16.34	942.61	195.14	179.06		375.20		545.33	396.28		226.16	$10_3 12{:}1_1$	12:1/10/10/10
Glc-40:0-Gly_2	11.74	973.61	169.12	179.06	161.05	349.19	623.43	519.32	453.30		283.17	10_4	10/10/10/10
30:0-Gly	9.31					187.13			396.28		226.14	10_3	10/10/10
36:0-Gly	13.62					159.10, 187.13			340.21, 368.24, 396.28		198.11, 226.15	$8_2 10_2$	non-specific
38:0-Gly	18.20					159.10, 187.13			368.24, 396.28		226.14	$8_1 10_3$	non-specific, 10_1Gly
	18.46					159.10, 187.13			368.24, 396.28		198.11	$8_1 10_3$	non-specific, 8_1Gly
40:0-Gly	23.03					187.13			396.28		226.14	10_4	10/10/10/10

Table S2 Detailed characterization of the identified species from pyruvate cultivation of *A. borkumensis* based on MS/MS. Characterization was performed for the individual retention times of the double peaks, where MS/MS could be recorded. The fragments detected in positive ion mode are listed, and the conclusions for the composition and position of the fatty acyl groups are summarized

**Species**	**Rt /min**	**$[M+Na]^+$**	**1 Glc**	**a FA_4Gly**	**b $GlcFA_1$**	**c FA_3Gly**	**d $GlcFA_2$**	**e FA_2Gly**	**f $GlcFA_3$**	**g FA_1Gly**	**Fatty acyl composition**	**Fatty acyl position**
Glc-34:0-Gly	3.75	856.50		694.45	345.15, 373.18	506.31, 534.34	459.22, 487.25, 515.28	364.21, 392.24	629.35, 657.38	222.11	$8_3 10_1$	non-specific
	4.05											No MS/MS
Glc-36:0-Gly	5.87	884.53	203.05	722.48	345.15, 373.18	534.34, 562.37	515.28, 543.31	392.24, 420.27	657.38, 685.41	250.14	$8_2 10_2$	non-specific
	6.52	884.53		722.48	317.12	590.40	487.25	420.27	657.38	250.14	$6_1 10_3$	6/10/10/10
Glc-38:0-Gly	8.95	912.56	203.05	750.51	373.18	562.37	515.28, 543.31	392.24, 420.27	685.41	250.14	$8_1 10_3$	10/8/10/10, 10/10/8/10
	9.29	912.56	203.05	750.51	345.15, 373.18	562.37, 590.40	515.28, 543.31	392.24, 420.27	685.41, 713.44	222.11, 250.14	$8_1 10_3$	**8/10/10/10,** 10/10/10/8
Glc-38:0-Ala	9.49	926.58		764.52	373.18	576.39	515.28, 543.31	406.26 434.29	685.41	264.16	$8_1 10_3$	10/8/10/10, 10/10/8/10
	9.88	926.58		764.52	345.15, 373.18	576.38, 604.42	515.28, 543.31	406.26 434.29	685.41, 713.44	264.16	$8_1 10_3$	**8/10/10/10,** 10/10/10/8
Glc-39:0-Gly	11.07	926.58		764.52	359.17, 373.18	576.39, 590.40	529.30, 543.31	406.26, 420.27	699.43		$9_1 10_3$	non-specific
Glc-40:0-Gly	13.29	940.60		778.54	373.18	590.40	543.31	420.27	713.44	250.14	10_4	10/10/10/10
Glc-40:0-Ala	13.83	954.61		792.56	373.18	604.42	543.31	434.29	713.44	264.16	10_4	10/10/10/10
Glc-42:0-Gly	18.13	968.63	203.05	806.57	373.18, 401.21	590.40, 618.43	543.31, 571.34	420.27, 448.30	713.44, 741.48	250.14, 278.17	$10_3 12_1$	10/10/10/12, 12/10/10/10
Glc-40:1-Gly	11.29											No MS/MS
Glc-42:1-Gly	15.92	966.61		804.55	373.18	616.42	543.31, 569.33	446.29	713.44, 739.46		$10_3 12{:}1_1$	non-specific, Glc10_1
	16.32											No MS/MS
Glc-40:0-Gly_2	11.75	997.62		835.56	373.18	647.42	543.31	477.29	713.44	307.16	10_4	10/10/10/10
30:0-Gly	9.24	608.41						420.27		250.14	10_3	10/10/10
36:0-Gly	13.61	722.48				534.34, 562.37		364.21, 392.24, 420.27		222.11	$8_2 10_2$	non-specific
38:0-Gly	18.18	750.51				562.37, 590.40		392.24, 420.27		250.14	$8_1 10_3$	non-specific, 10_1Gly
	18.43											No MS/MS
40:0-Gly	23.02	778.54				590.40		420.27		250.14	10_4	10/10/10/10

Table S3 Detailed characterization of the identified species from hexadecane cultivation of *A. borkumensis* based on MS/MS. Characterization was performed for the individual retention times of the double peaks, where MS/MS could be recorded. The fragments detected in negative ion mode are listed, and the conclusions for the composition and position of the fatty acyl groups are summarized

**Species**	**Rt /min**	**[M-H]$^-$**	**FS**	**1 Glc**	**2 Glc (-H_2O)**	**b $GlcFA_1$**	**c FA_3Gly**	**d $GlcFA_2$**	**e FA_2Gly**	**f $GlcFA_3$**	**g FA_1Gly**	**Fatty acyl composition**	**Fatty acyl position**
Glc-36:0-Gly	5.94	860.53	141.09, 169.12	179.06		321.16, 349.19		463.25, 491.28, 519.32	340.21, 368.24, 396.28		198.11, 226.14	$8_2 10_2$	non-specific
	6.57	860.53	113.06	179.06		293.12	509.58	463.25	340.21, 396. 28	633.39	226.14	$6_1 10_3$	6/10/10/10
Glc-38:0-Gly	9.02	888.57	141.09 169.12	179.06	161.05	349.19	538.38	491.29, 519.32	368.24, 396.28	661.42	226.14	$8_1 10_3$	10/8/10/10, 10/10/8/10
	9.32	888.57	141.09, 169.12	179.06	161.05	321.16, 349.19		491.29, 519.32	368.24, 396.28	661.41, 689.45	198.11, 226.14	$8_1 10_3$	**8/10/10/10**, 10/10/10/8
Glc-39:0-Gly	11.09	902.58	155.11, 169.12	179.06	161.05	335.17 349.19	552.39	505.30, 519.32	382.26, 396.28	675.43	212.13, 226.14	$9_1 10_3$	non-specific
Glc-40:0-Gly	13.28	916.60	169.12	179.06	161.05	349.19	566.40	519.32	396.28	689.44	226.14	10_4	10/10/10/10
Glc-42:0-Gly	18.17	944.63	169.12, 197.15	179.06	161.05	349.19, 377.22	566.41	519.32, 547.35	396.28, 424.31	689.45, 717.48	226.14, 254.18	$10_3 12_1$	10/10/10/12, 12/10/10/10
Glc-40:1-Gly	11.29	914.58	167.11, 169.12	179.06	161.04	349.19		517.30, 519.32	394.26 396.28	687.43	224.13 226.14	$10_3 10{:}1_1$	non-specific, $Glc10_1$
Glc-42:1-Gly	15.98	942.62	169.12	179.06	161.04	349.19		519.32, 545.33	396.27, 422.29		226.14, 252.16	$10_3 12{:}1_1$	non-specific, $Glc10_1$
Glc-40:0-Gly_2	11.81	973.62	169.12	179.06	161.05	349.19	623.43	519.32	453.30	689.45	283.17	10_4	10/10/10/10
30:0-Gly	9.34					187.13			396.28		226.14	10_3	10/10/10
38:0-Gly	18.21					159.10, 187.13			368.24, 396.28		226.14	$8_1 10_3$	non-specific, 10_1Gly
40:0-Gly	23.06					187.13			396.28		226.14	10_4	10/10/10/10
42:0-Gly	27.67					187.13, 215.17			396.28, 424.31		226.14, 254.18	$10_3 12_1$	non-specific

Table S4 Detailed characterization of the identified species from hexadecane cultivation of *A. borkumensis* based on MS/MS. Characterization was performed for the individual retention times of the double peaks, where MS/MS could be recorded. The fragments detected in positive ion mode are listed, and the conclusions for the composition and position of the fatty acyl groups are summarized

Species	Rt /min	$[M+Na]^+$	1 Glc	a FA_4Gly	b $GlcFA_1$	c FA_3Gly	d $GlcFA_2$	e FA_2Gly	f $GlcFA_3$	g FA_1Gly	Fatty acyl composition	Fatty acyl position
Glc-36:0-Gly	5.90											No MS/MS
	6.50											No MS/MS
Glc-38:0-Gly	8.96	912.56	203.05	750.51	373.18	562.37	515.28, 543.31	392.24, 420.27	685.41	250.14	$8_1 10_3$	10/8/10/10, 10/10/8/10
	9.27	912.56	203.05	750.51	345.15, 373.18	562.37, 590.40	515.28, 543.31	392.24, 420.27	685.41, 713.44	222.11, 250.14	$8_1 10_3$	**8/10/10/10,** 10/10/10/8
Glc-39:0-Gly	11.03	926.58		764.53	359.17, 373.18	576.39, 590.40	529.30, 543.31	406.26, 420.27	699.43, 713.45		$9_1 10_3$	non-specific
Glc-40:0-Gly	13.29	940.60		778.54	373.18	590.40	543.31	420.27	713.44	250.14	10_4	10/10/10/10
Glc-42:0-Gly	18.14	968.63	203.05	806.58	373.18, 401.21	590.40, 618.43	543.31, 571.34	420.27, 448.30	713.44, 741.47	250.14, 278.17	$10_3 12_1$	10/10/10/12, 12/10/10/10
Glc-40:1-Gly	11.20											No MS/MS
Glc-42:1-Gly	15.93	966.61			373.18	616.42	543.31, 569.33	446.29	713.44, 739.46		$10_3 12{:}1_1$	non-specific, $Glc10_1$
Glc-40:0-Gly_2	11.75	997.62		835.56	373.18	647.42	543.31	477.29	713.44	307.16	10_4	10/10/10/10
30:0-Gly	9.27	608.41						420.27		250.14	10_3	10/10/10
38:0-Gly	18.15	750.51				562.37, 590.40		392.24, 420.27		250.14	$8_1 10_3$	non-specific, 10_1Gly
40:0-Gly	23.04	778.54				590.40		420.27		250.14	10_4	10/10/10/10
42:0-Gly	27.69	806.58				590.40, 618.43		420.27, 448.30		250.14, 278.17	$10_3 12_1$	non-specific

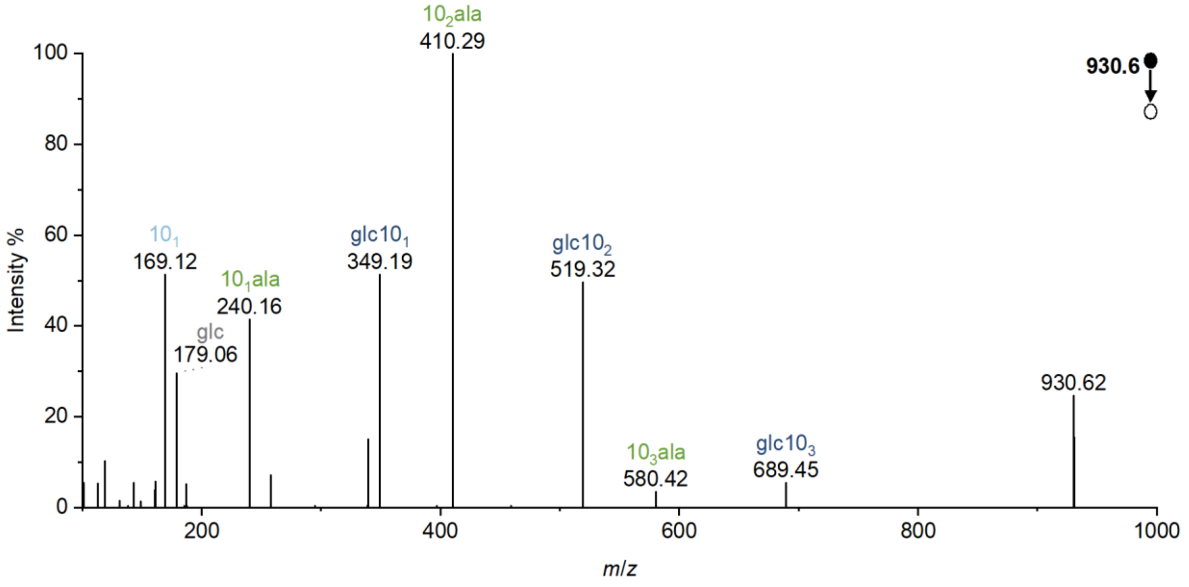

Fig. S2 Fragmentation of the postulated species Glc-40:0-Ala eluting at a retention time of 13.82 min in negative ion mode. MS/MS spectrum for the precursor *m/z* 930.6 ($[M-H]^-$) acquired with HCD at NCE 18 in the pyruvate culture extract. For a discussion of the observed fragments, see section 3.2

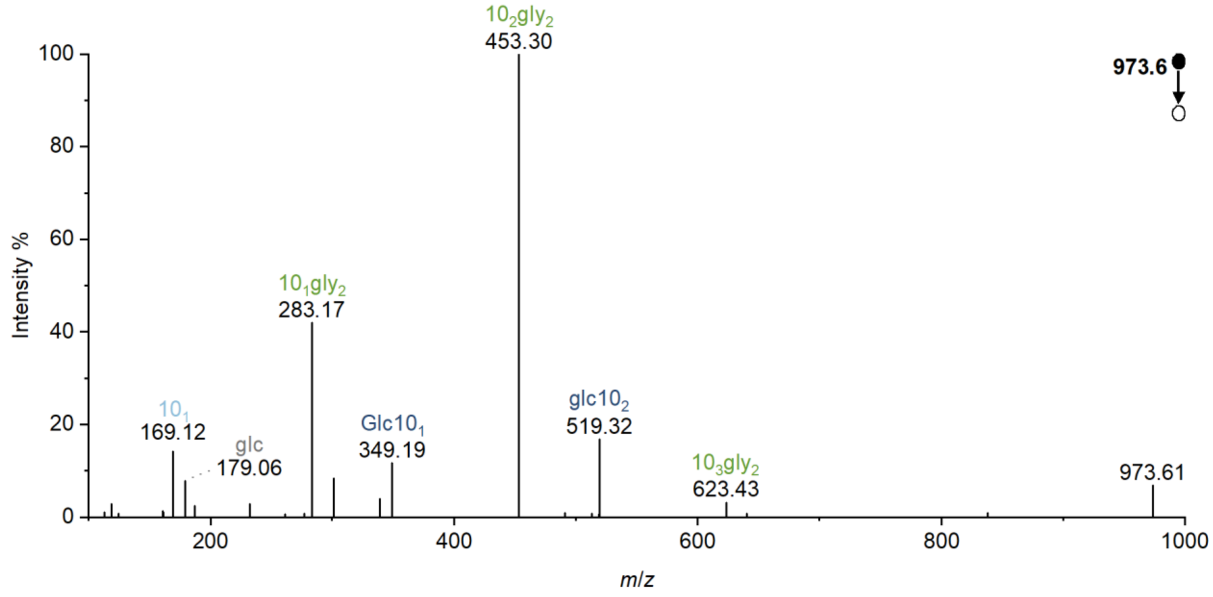

Fig. S3 Fragmentation of the postulated species Glc-40:0-Gly$_2$ eluting at a retention time of 11.71 min in negative ion mode. MS/MS spectrum for the precursor *m/z* 973.6 ([M-H]$^-$) acquired with HCD at NCE 18 in the pyruvate culture extract. For a discussion of the observed fragments, see section 3.2

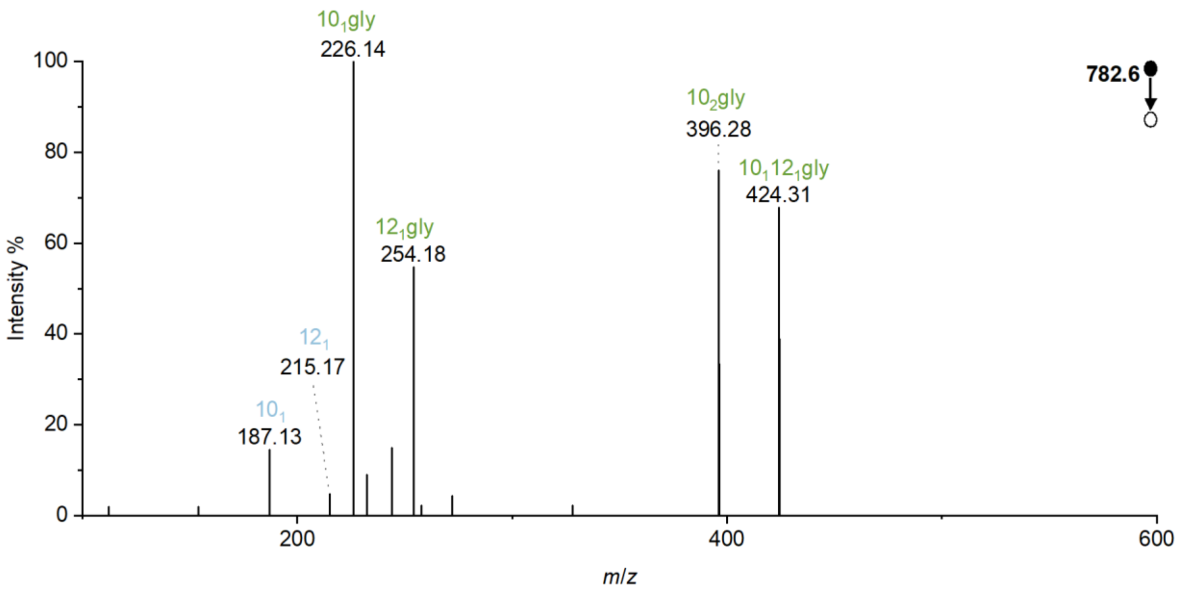

Fig. S4 Fragmentation of aglycones in negative ion mode at the example of 42:0-Gly eluting at a retention time of 27.71 min. MS/MS spectrum for *m/z* 782.6 ([M-H]$^-$) acquired with HCD at NCE 18 for the extract from hexadecane cultivation. For a discussion of the observed fragments, see section 3.2

MS/MS spectra for all identified species in negative ion mode (raw data).

For the different *m*/*z*, the EIC extracted with boxcar smoothing over 3 points and 5 ppm mass tolerance and selected MS/MS spectra for the sodium adduct are shown.

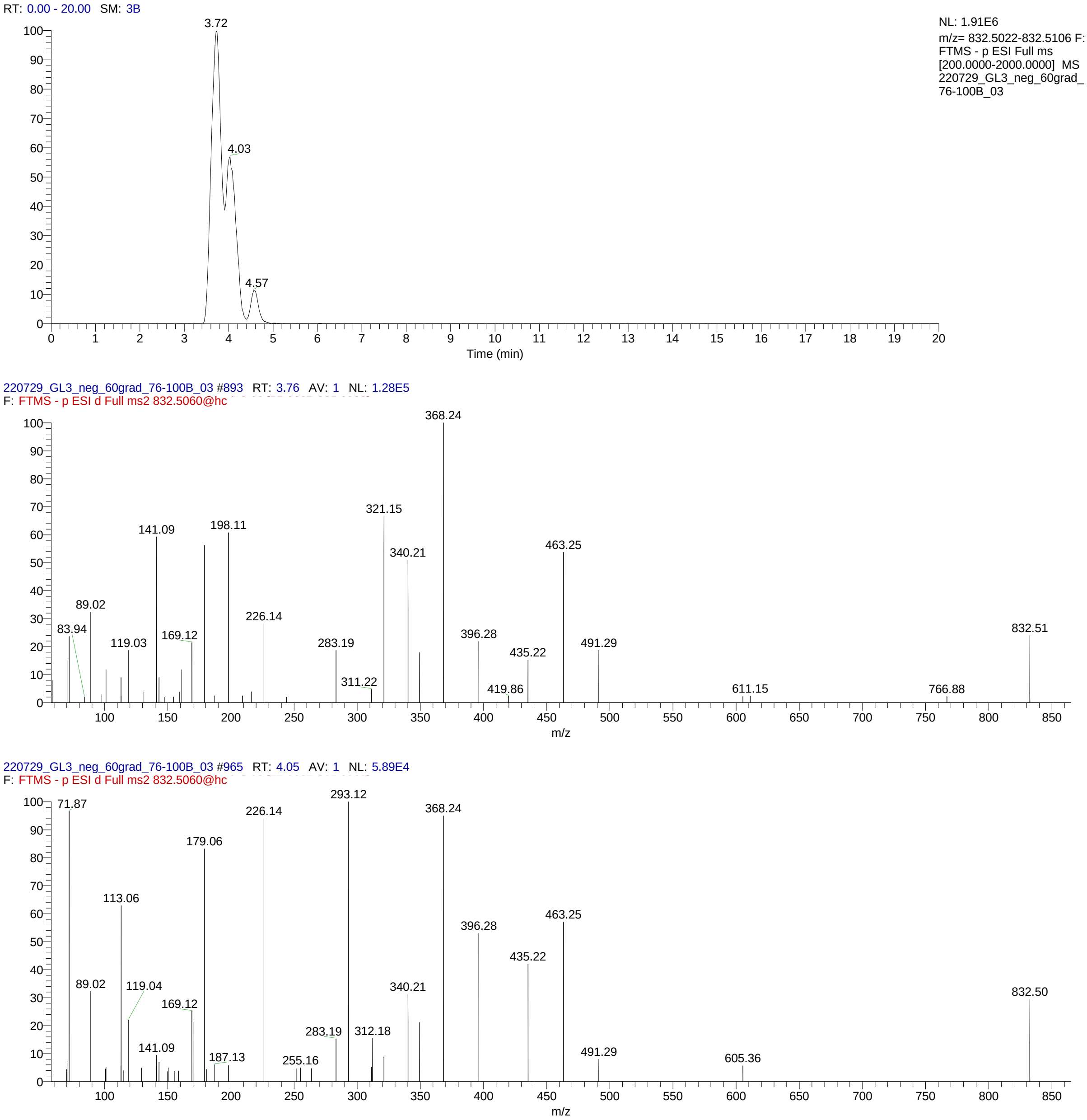

Fig. S5 MS/MS of Glc-34:0-Gly (*m*/*z* 832.5, $[M-H]^-$) in negative ion mode at 3.76 min and 4.05 min at NCE 18 together with the EIC. Measurement of the pyruvate culture extract

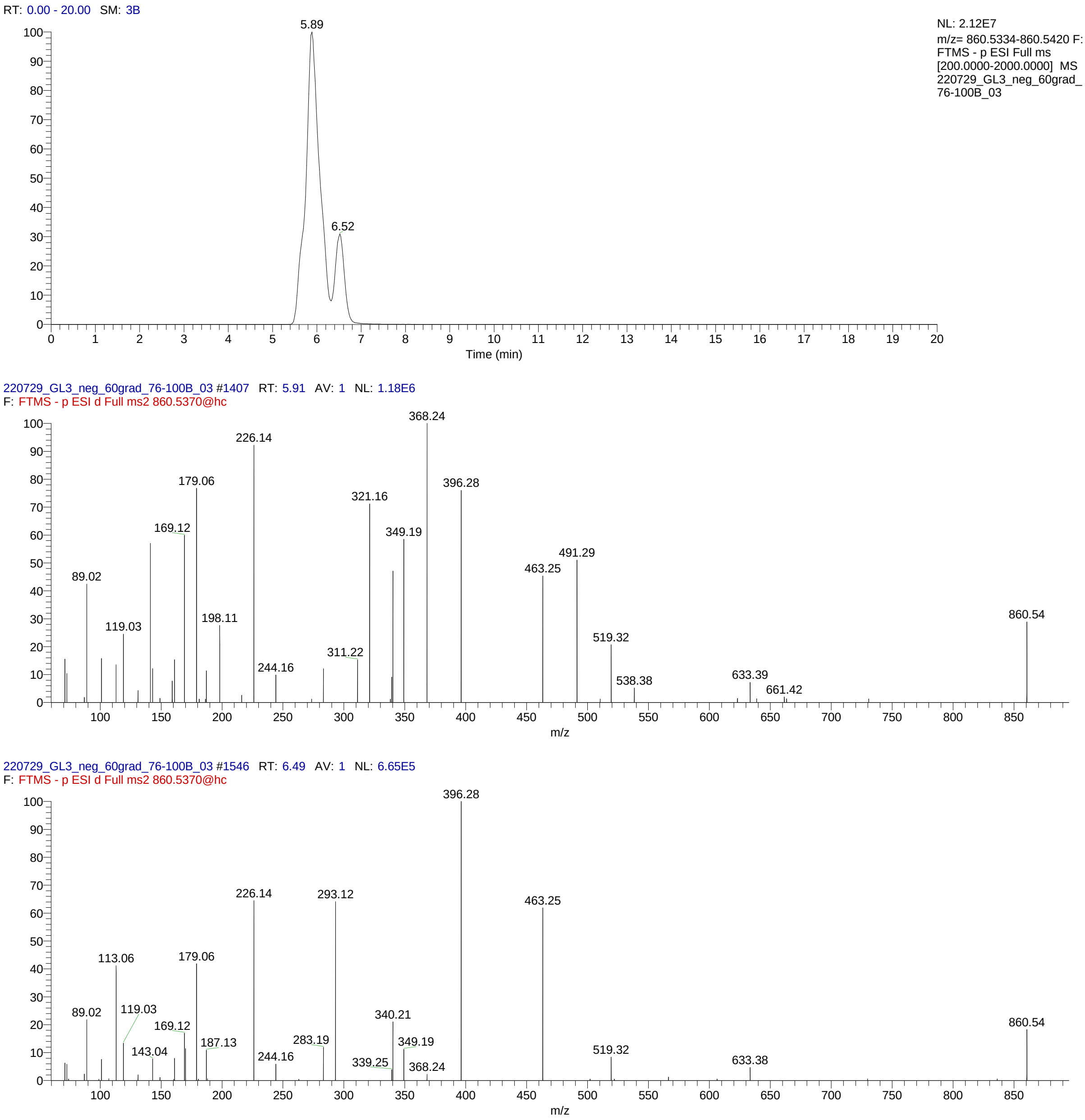

Fig. S6 MS/MS of Glc-36:0-Gly (*m/z* 860.5, $[M-H]^-$) in negative ion mode at 5.91 min and 6.49 min at NCE 18 together with the EIC. Measurement of the pyruvate culture extract

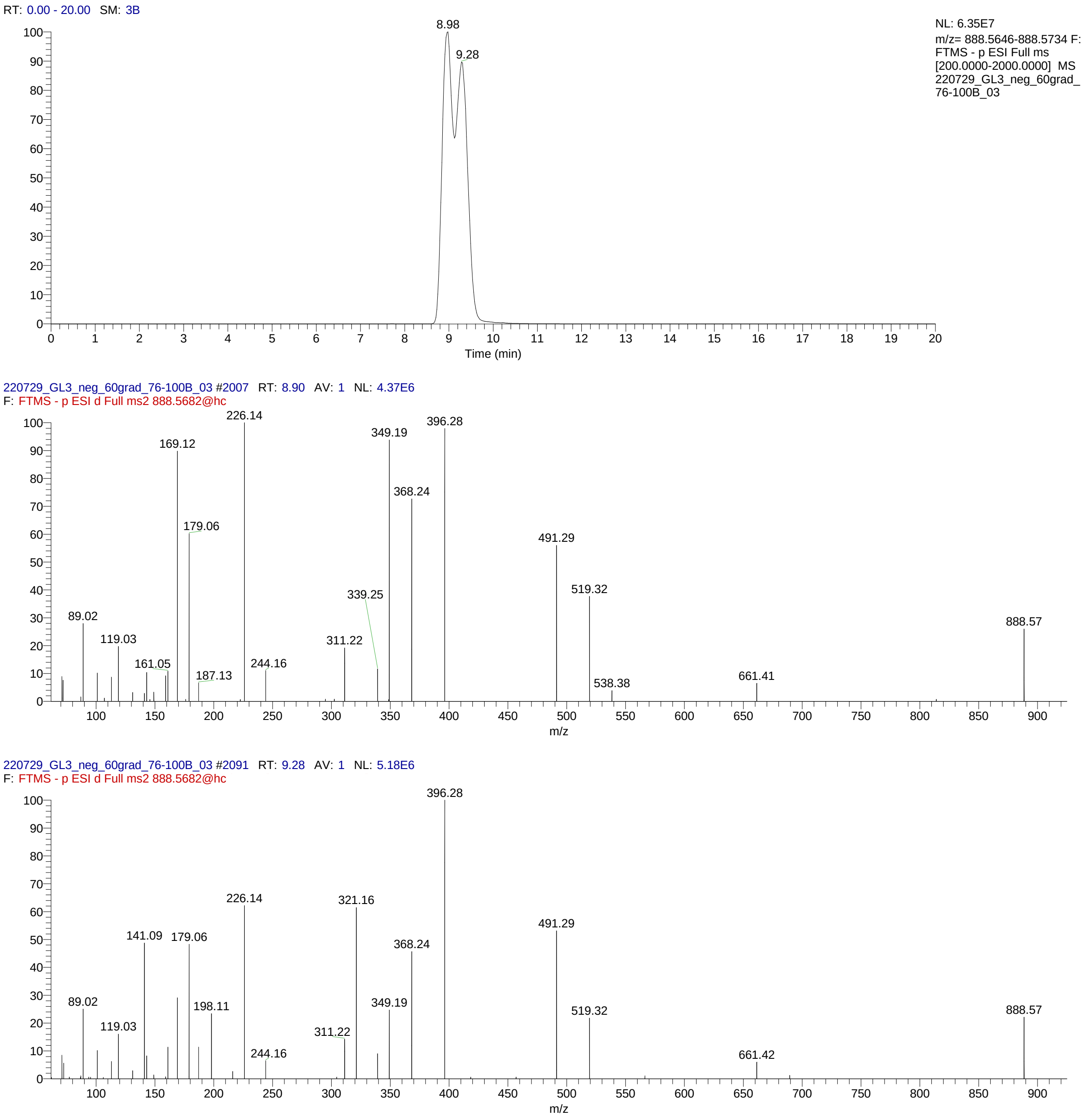

Fig. S7 MS/MS of Glc-38:0-Gly (*m/z* 888.6, [M-H]-) in negative ion mode at 8.90 min and 9.28 min at NCE 18 together with the EIC. Measurement of the pyruvate culture extract

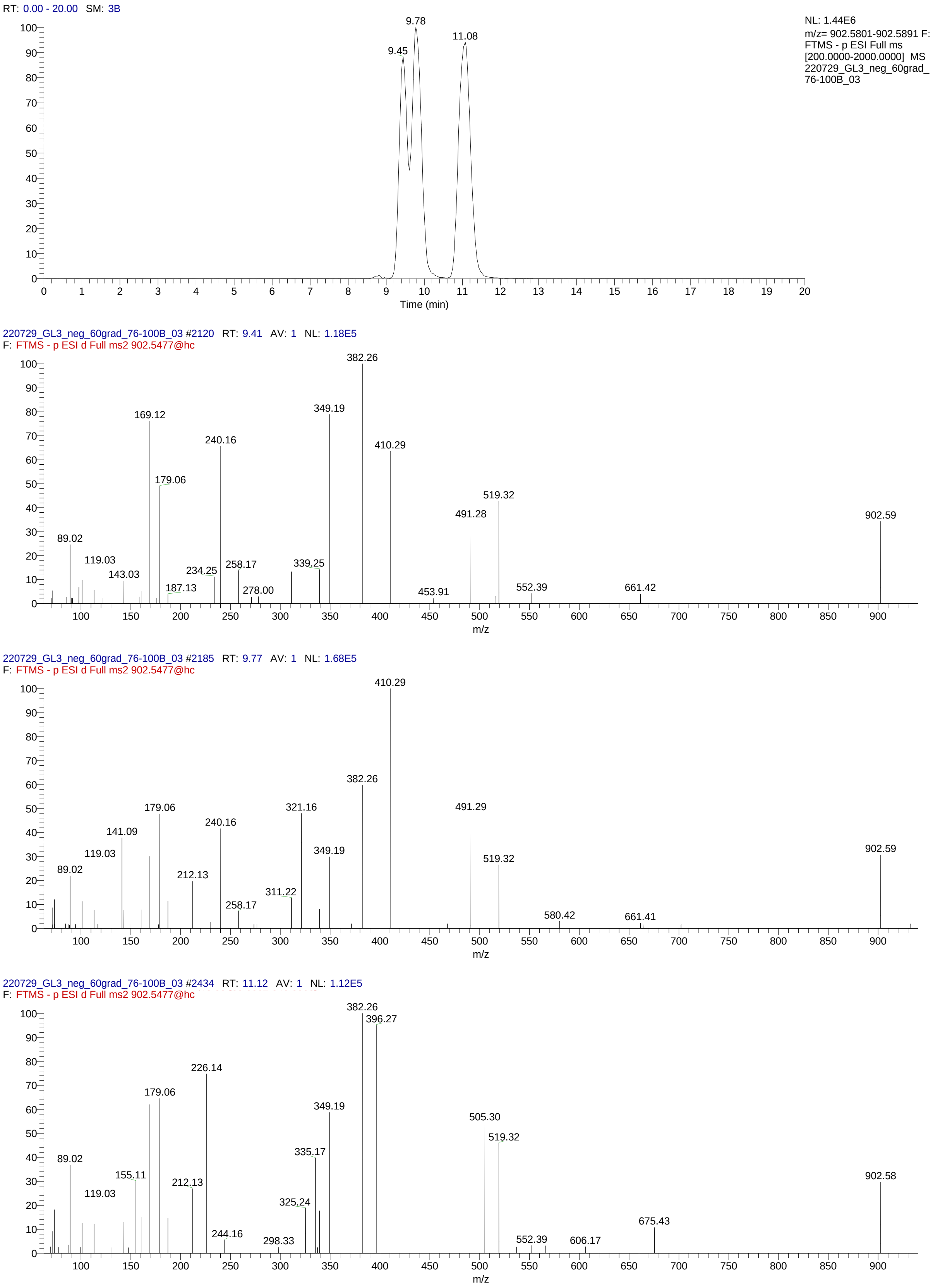

Fig. S8 MS/MS of Glc-38:0-Ala/Glc-39:0-Gly (*m/z* 902.6, [M-H]-) in negative ion mode at 9.41 min, 9.77 min, and 11.12 min at NCE 18 together with the EIC. Measurement of the pyruvate culture extract

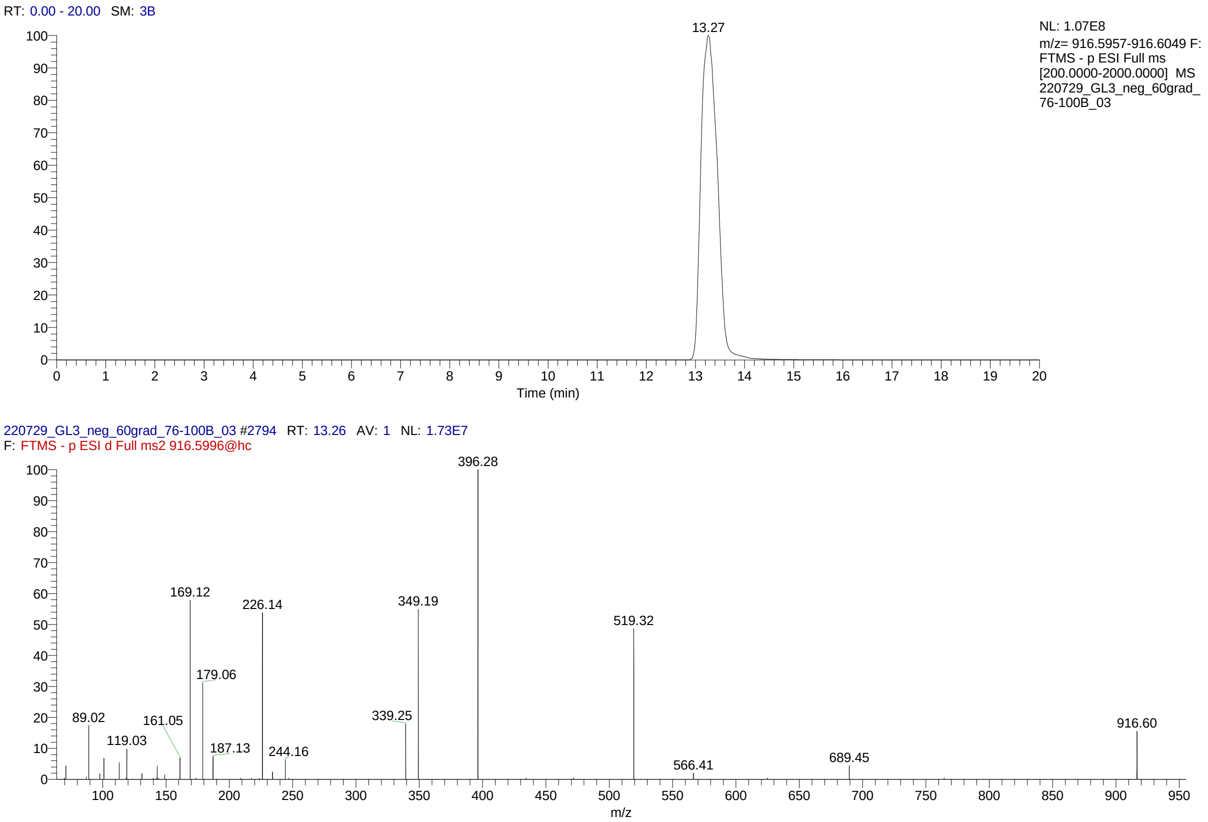

Fig. S9 MS/MS of Glc-40:0-Gly (*m/z* 916.6, [M-H]-) in negative ion mode at 13.26 min at NCE 18 together with the EIC. Measurement of the pyruvate culture extract

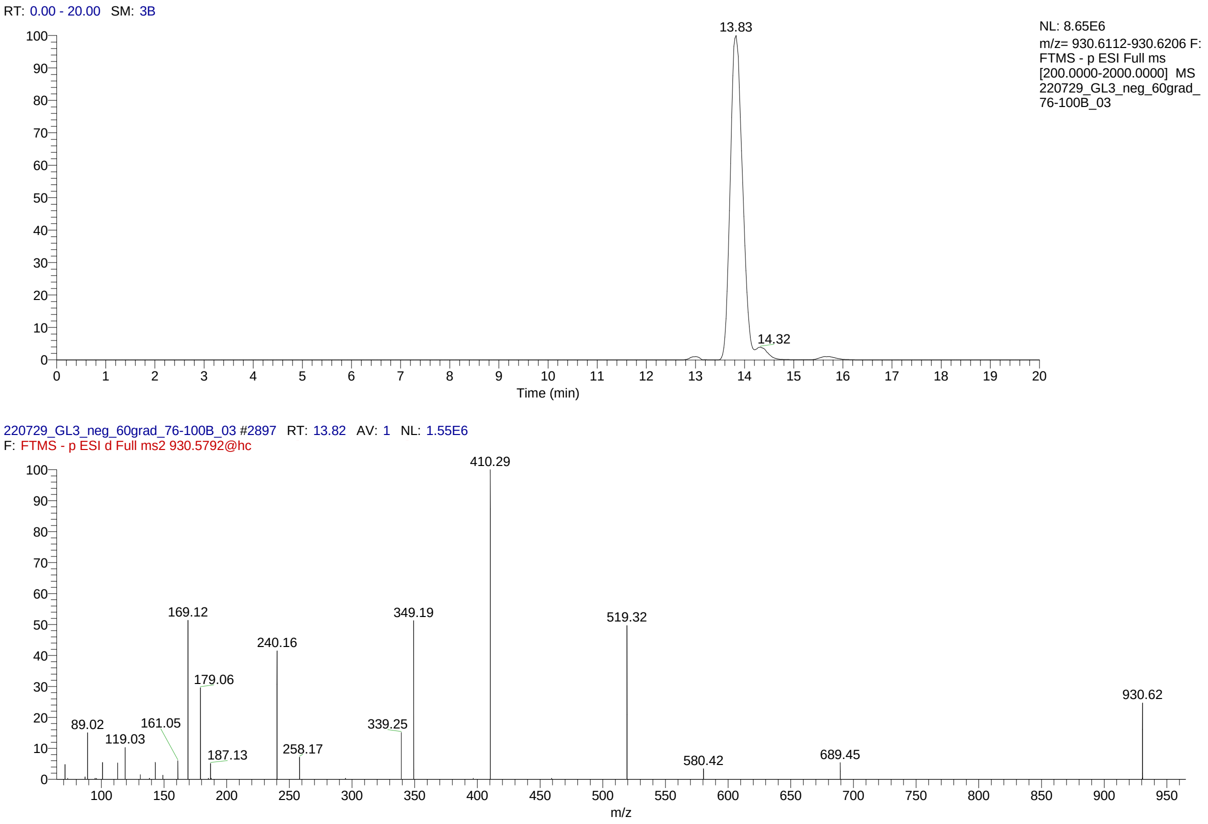

Fig. S10 MS/MS of Glc-40:0-Ala (*m/z* 930.6, [M-H]-) in negative ion mode at 13.82 min at NCE 18 together with the EIC. Measurement of the pyruvate culture extract

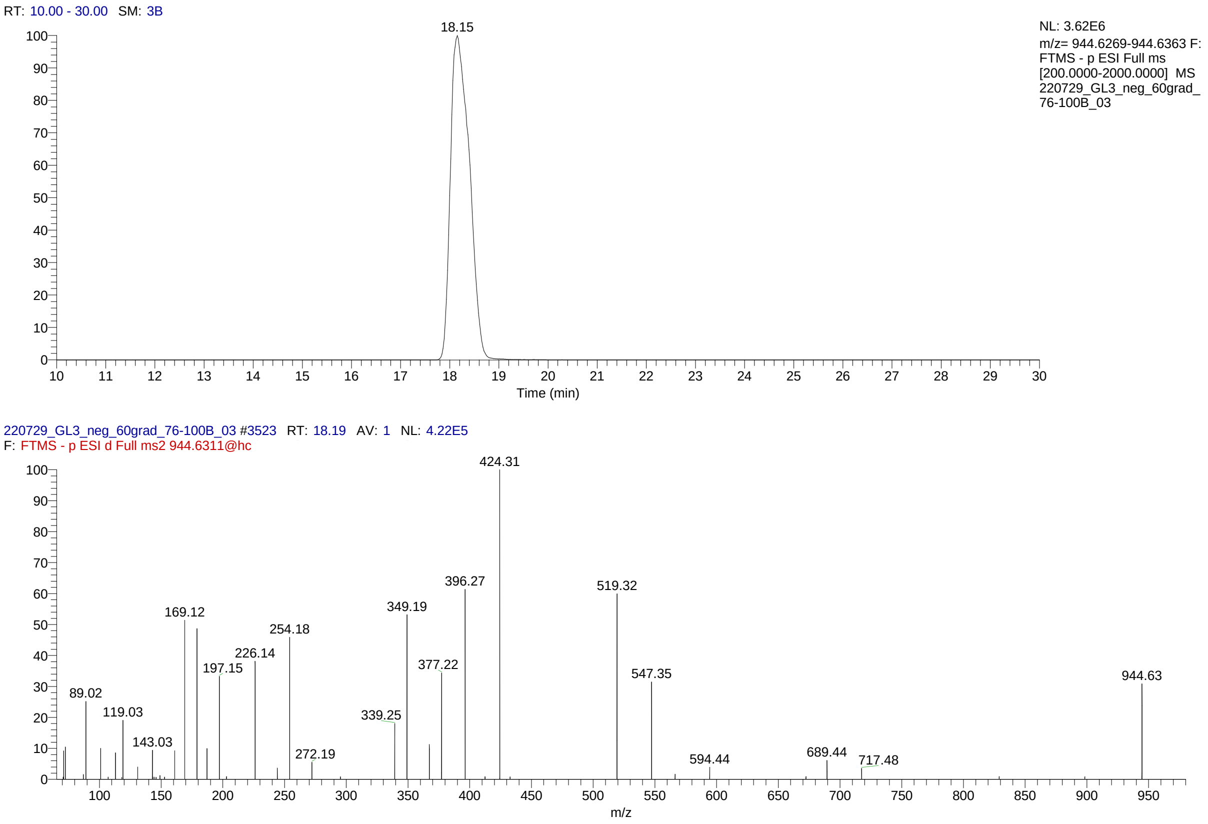

Fig. S11 MS/MS of Glc-42:0-Gly (*m/z* 944.6, [M-H]-) in negative ion mode at 18.19min at NCE 18 together with the EIC. Measurement of the pyruvate culture extract

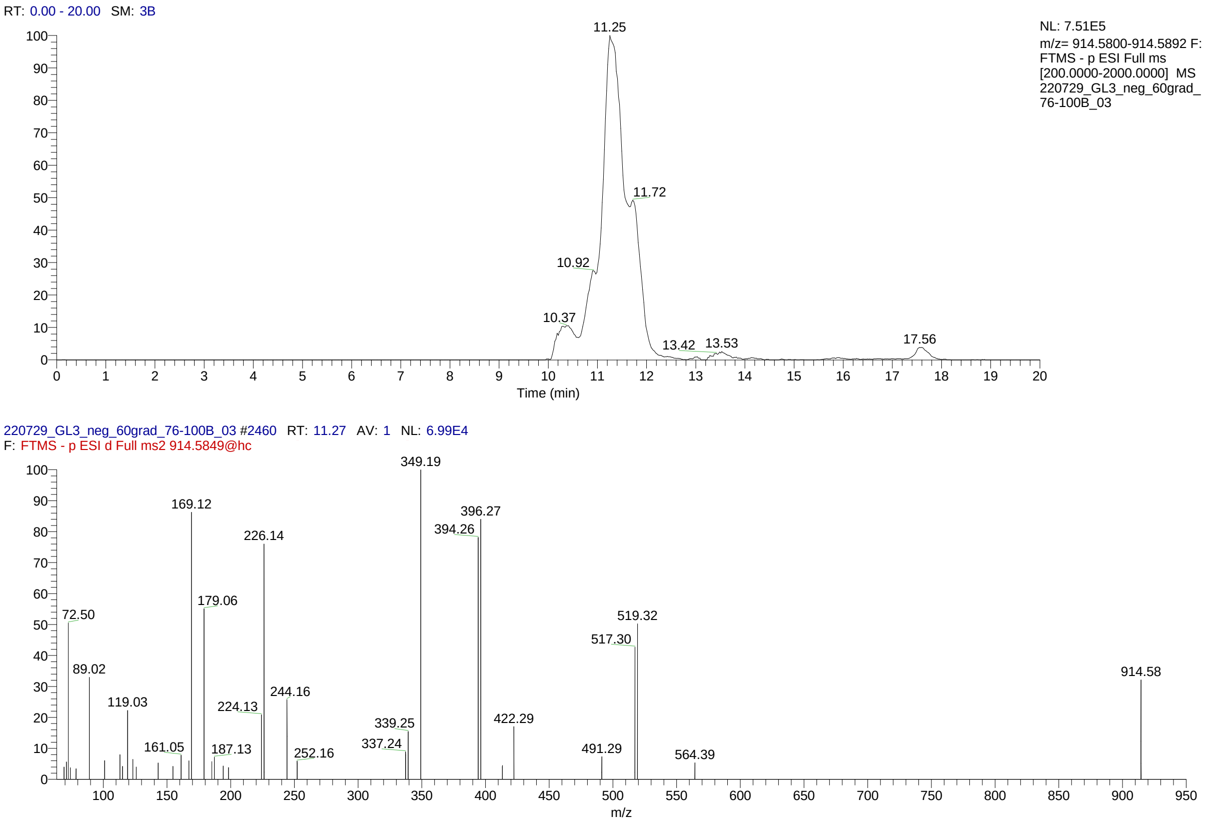

Fig. S12 MS/MS of Glc-40:1-Gly (*m/z* 914.6, [M-H]-) in negative ion mode at 11.27 min at NCE 18 together with the EIC. Measurement of the pyruvate culture extract

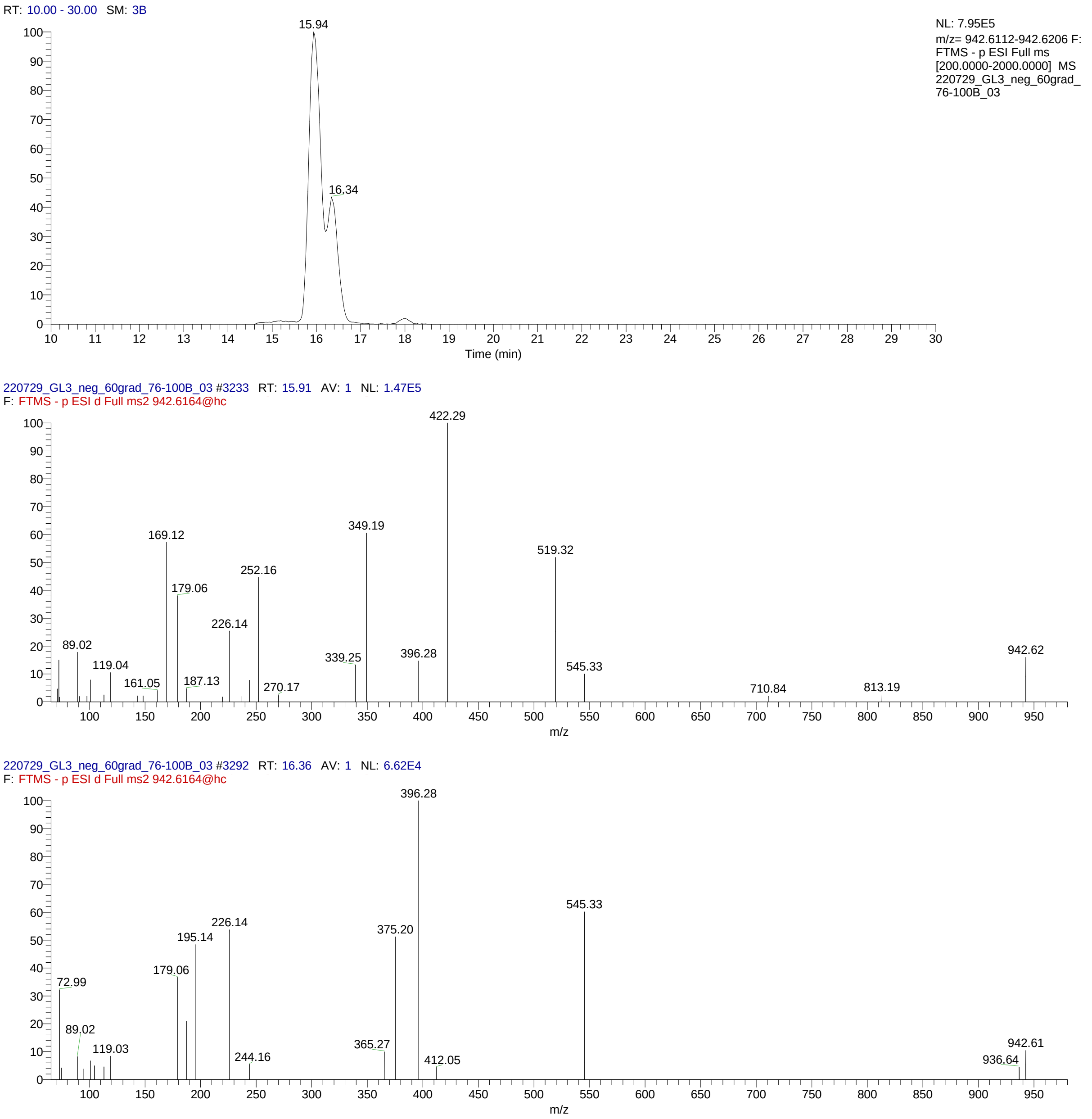

Fig. S13 MS/MS of Glc-42:1-Gly (*m/z* 942.6, [M-H]-) in negative ion mode at 15.91 min and 16.36 min at NCE 18 together with the EIC. Measurement of the pyruvate culture extract

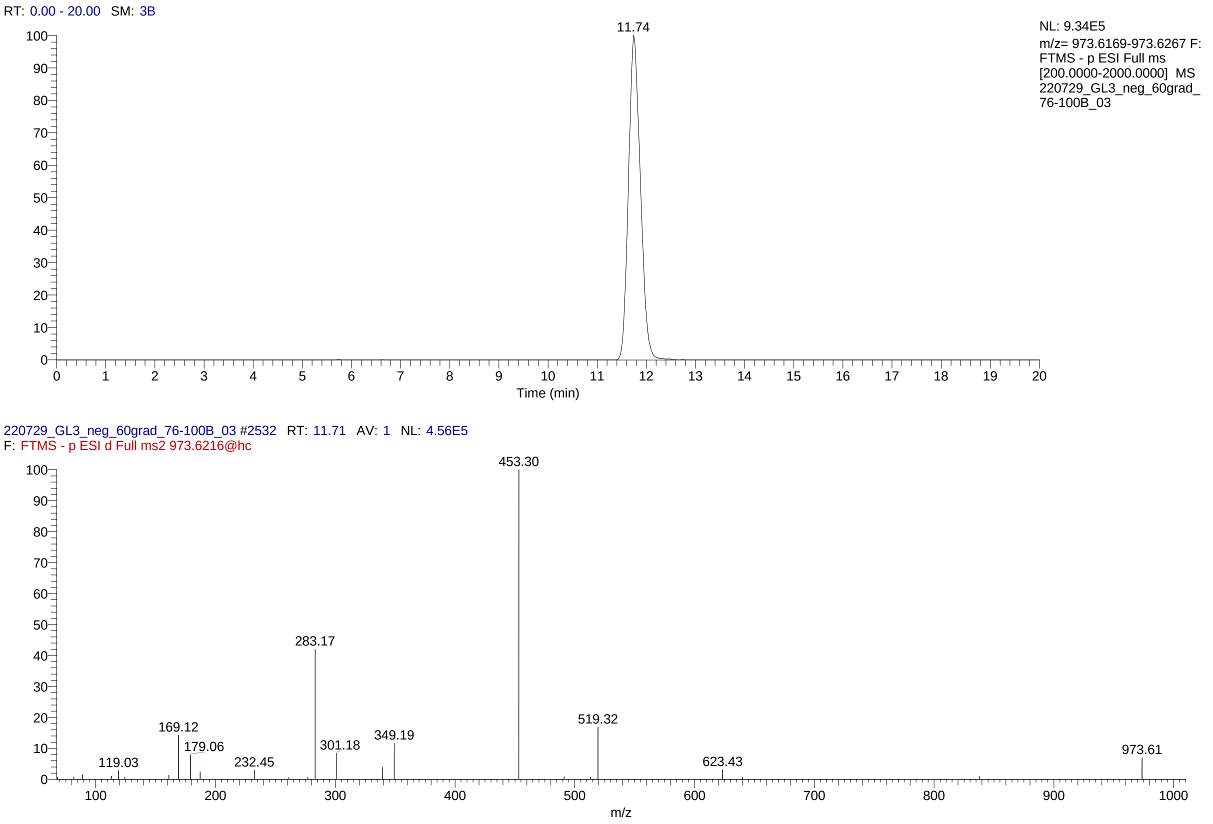

Fig. S14 MS/MS of Glc-40:0-Gly$_2$ (*m/z* 973.6, [M-H]-) in negative ion mode at 17.71 min at NCE 18 together with the EIC. Measurement of the pyruvate culture extract

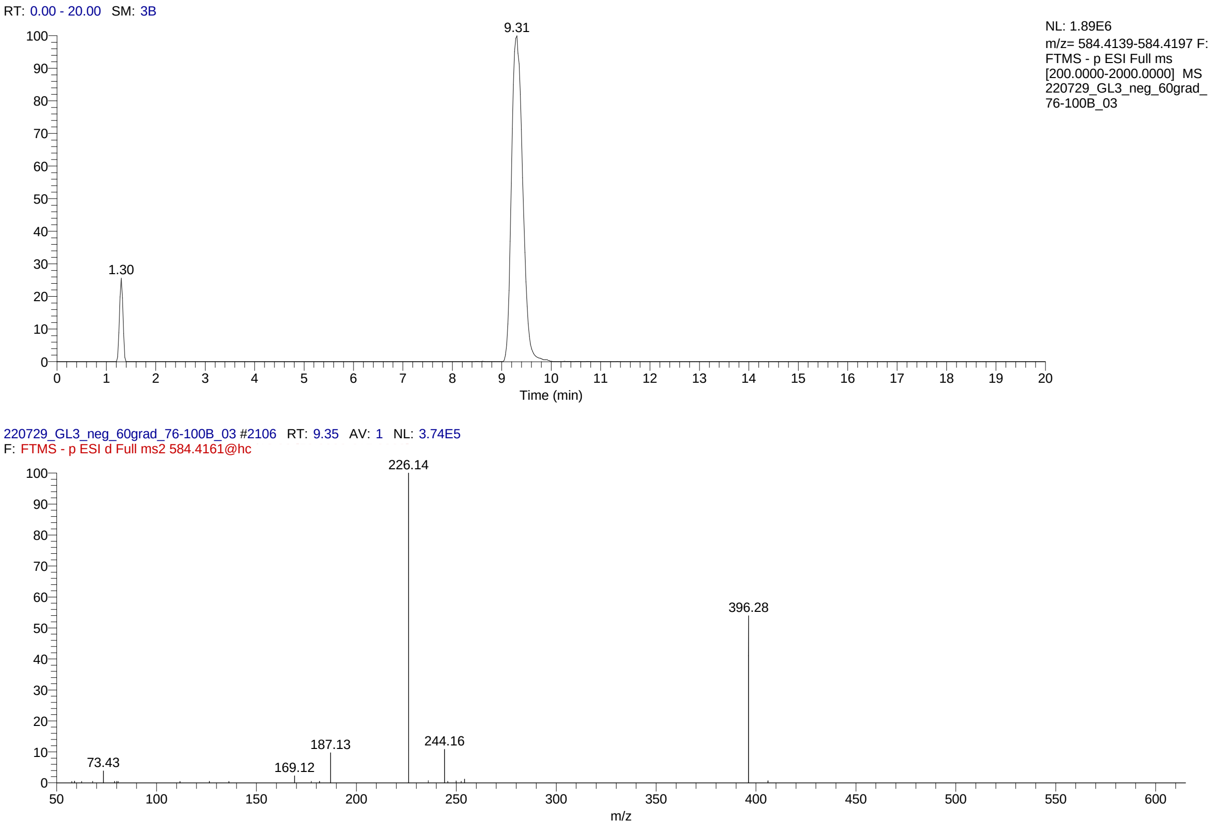

Fig. S15 MS/MS of 3×10:0-Gly (*m*/*z* 584.4, [M-H]-) in negative ion mode at 9.35 min at NCE 18 together with the EIC. Measurement of the pyruvate culture extract

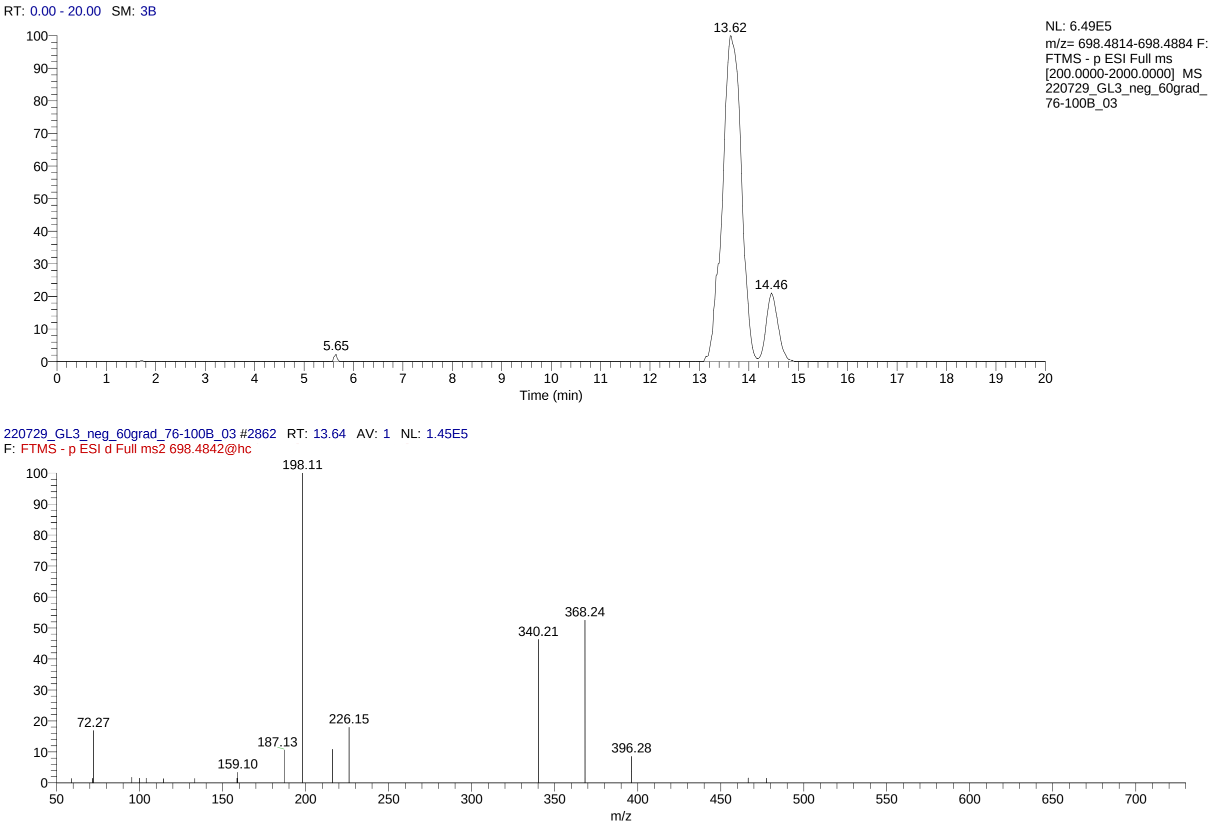

Fig. S16 MS/MS of 36:0-Gly (*m/z* 698.5, [M-H]-) in negative ion mode at 13.64 min at NCE 18 together with the EIC. Measurement of the pyruvate culture extract

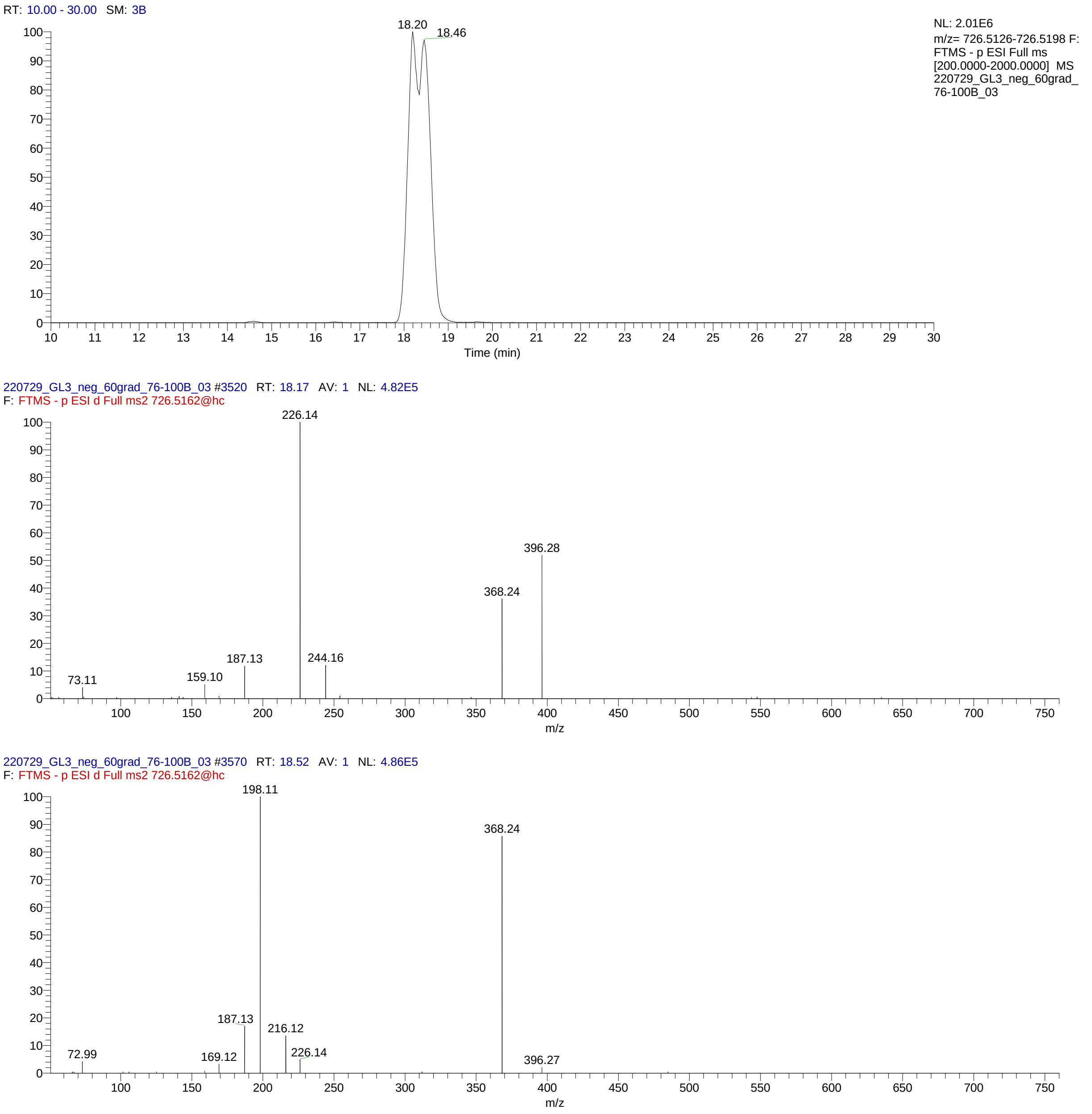

Fig. S17 MS/MS of 38:0-Gly (*m/z* 726.5, [M-H]-) in negative ion mode at 18.17 min and 18.52 min at NCE 18 together with the EIC. Measurement of the pyruvate culture extract

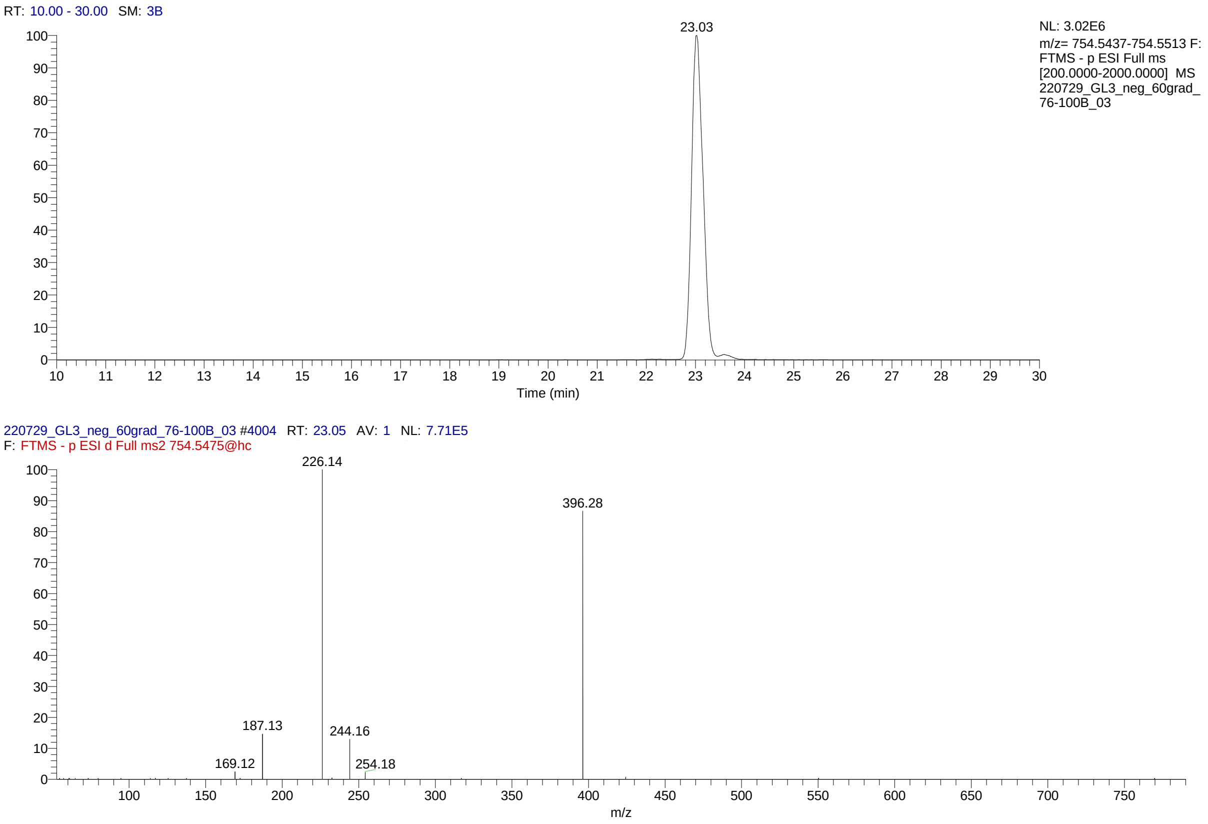

Fig. S18 MS/MS of 40:0-Gly (*m/z* 754.5, [M-H]-) in negative ion mode at 23.05 min at NCE 18 together with the EIC. Measurement of the pyruvate culture extract

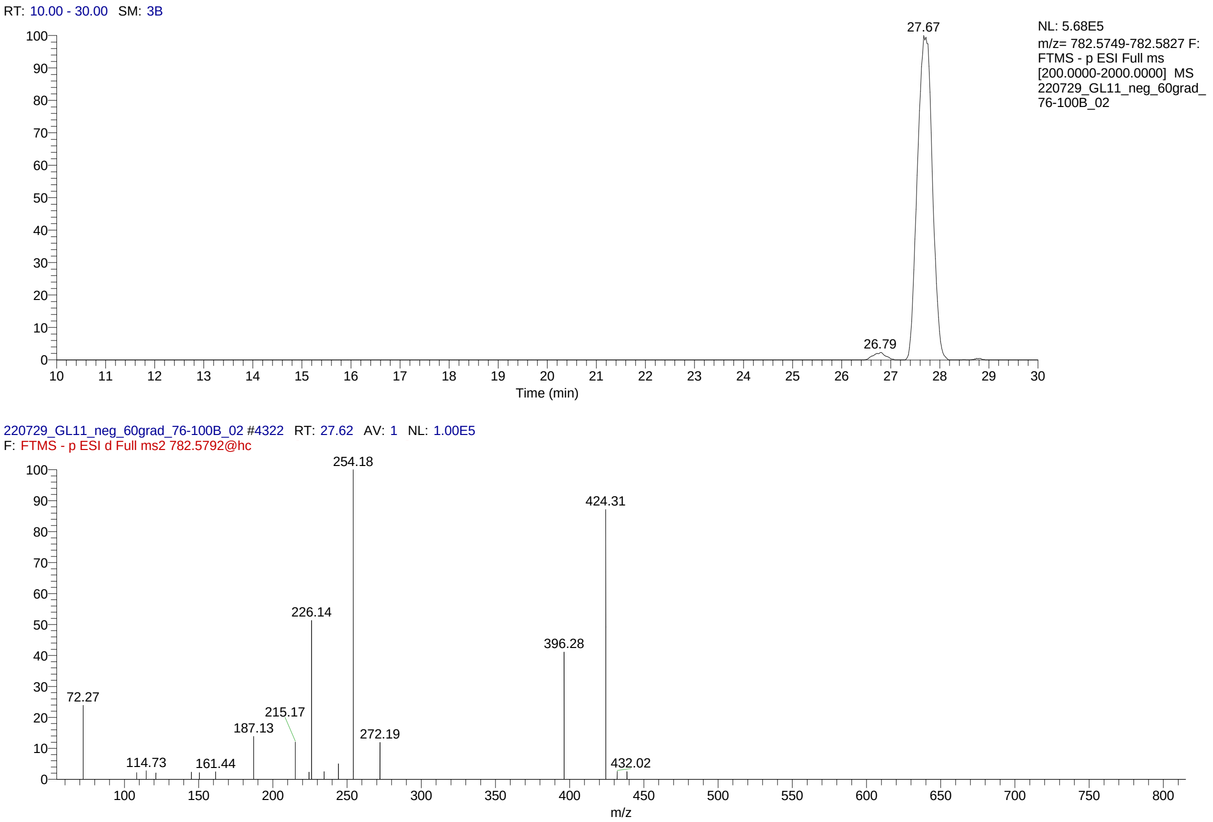

Fig. S19 MS/MS of 42:0-Gly (*m/z* 782.6, [M-H]-) in negative ion mode at 27.62 min at NCE 18 together with the EIC. Measurement of the hexadecane culture extract

MS/MS spectra for all identified species in positive ion mode (raw data).

For the different *m*/*z*, the EIC extracted with boxcar smoothing over 3 points and 5 ppm mass tolerance and selected MS/MS spectra for the sodium adduct are shown.

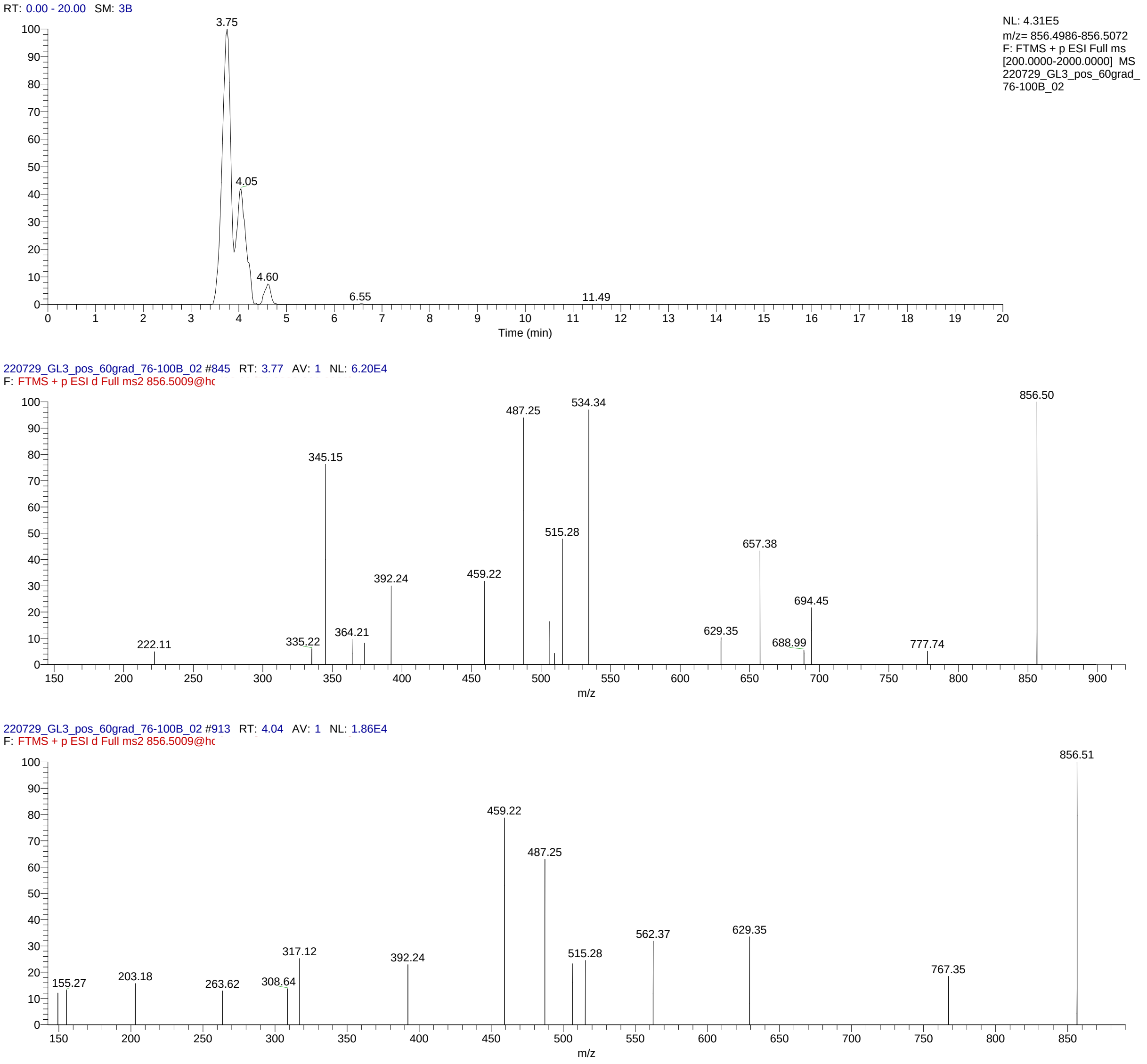

Fig. S20 MS/MS of Glc-34:0-Gly (*m*/*z* 856.5, $[M+Na]^+$) in positive ion mode at 3.77 min and 4.04 min at NCE 30 together with the EIC. Measurement of the pyruvate culture extract.

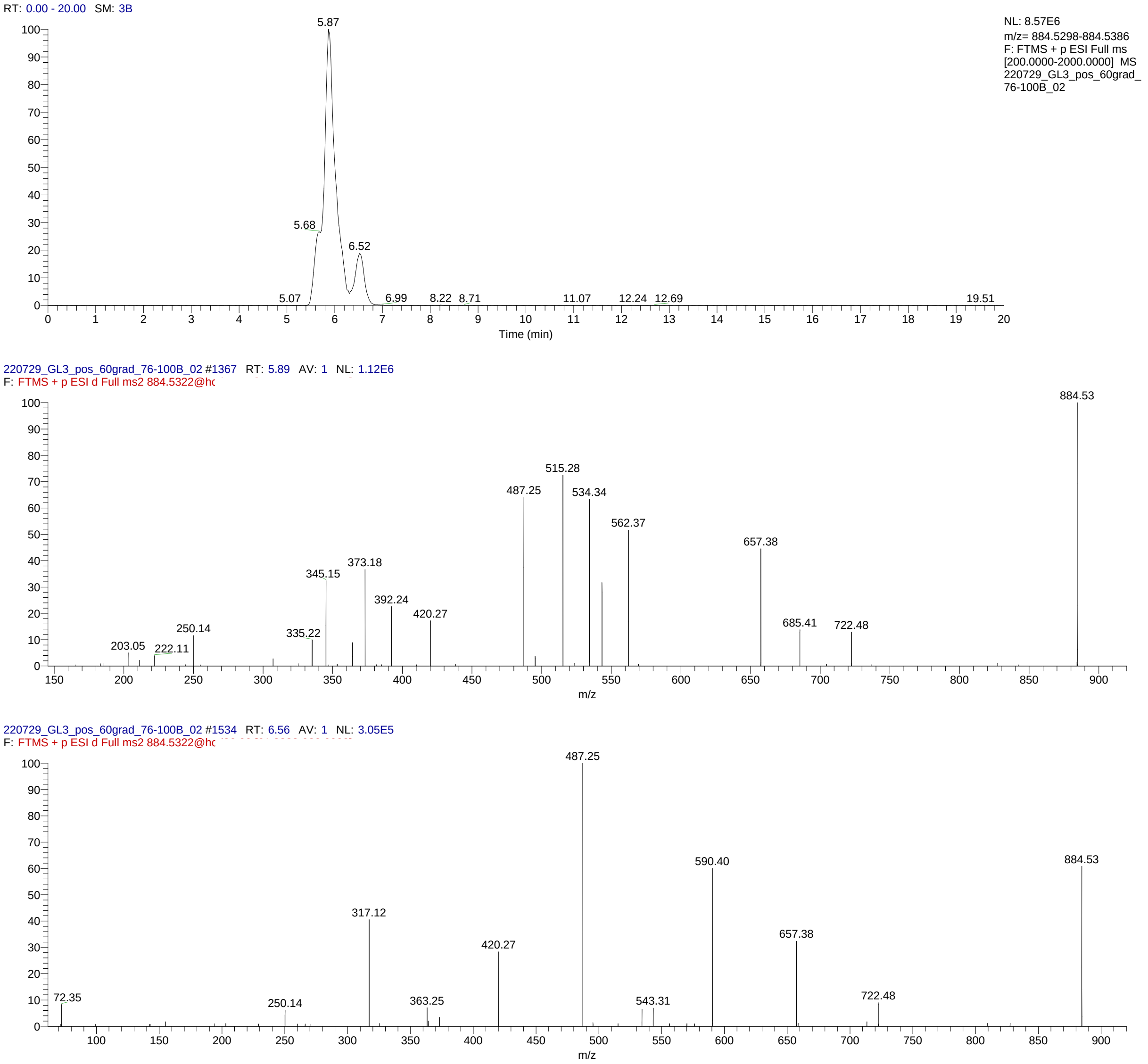

Fig. S21 MS/MS of Glc-36:0-Gly (*m*/*z* 884.5, $[M+Na]^+$) in positive ion mode at 5.89 min and 6.56 min at NCE 30 together with the EIC. Measurement of the pyruvate culture extract

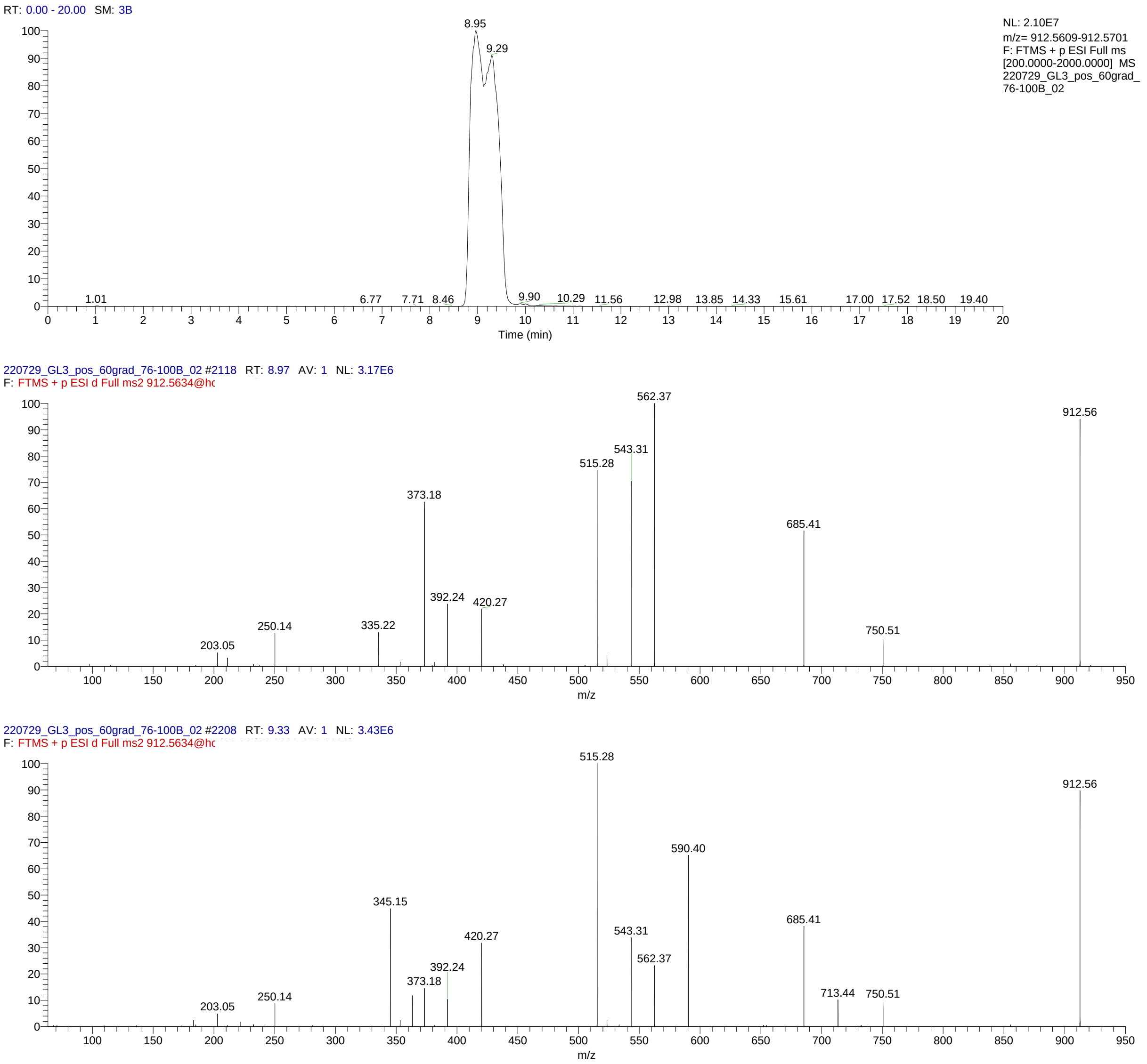

Fig. S22 MS/MS of Glc-38:0-Gly (*m*/*z* 912.6, $[M+Na]^+$) in positive ion mode at 8.97 min and 9.33 min at NCE 30 together with the EIC. Measurement of the pyruvate culture extract

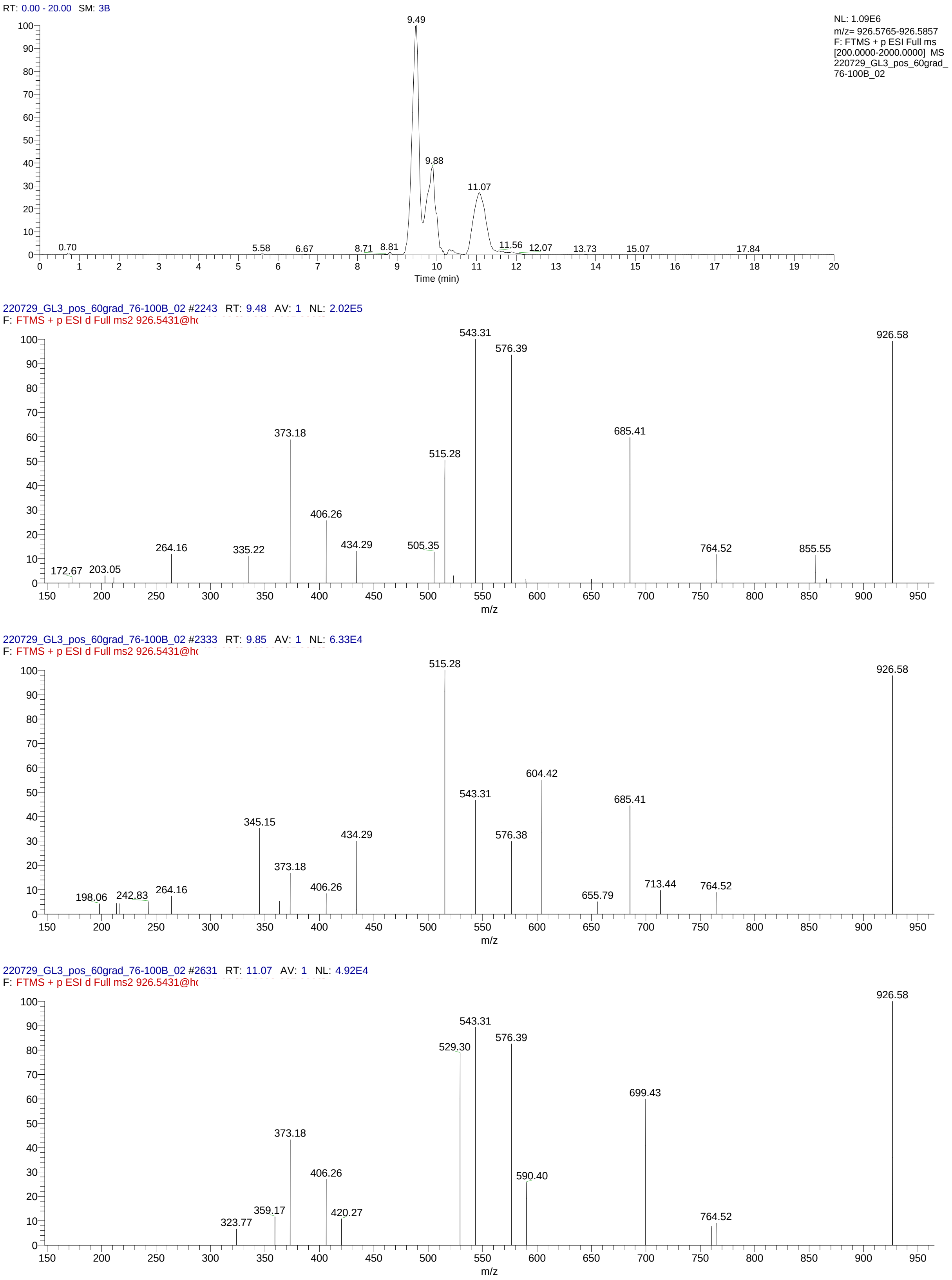

Fig. S23 MS/MS of Glc-38:0-Ala/Glc-39:0-Gly (*m*/*z* 926.6, $[M+Na]^+$) in positive ion mode at 9.48 min, 9.85 min, and 11.07 min at NCE 30 together with the EIC. Measurement of the pyruvate culture extract

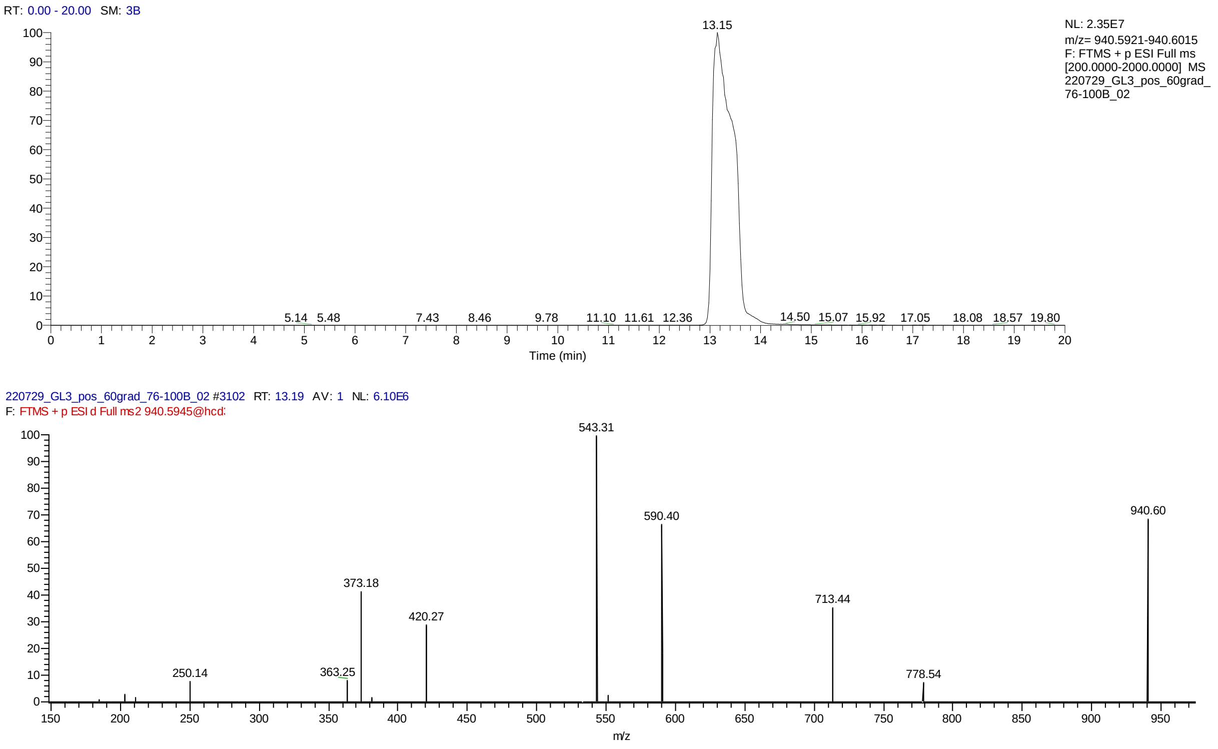

Fig. S24 MS/MS of Glc-40:0-Gly (*m/z* 940.6, $[M+Na]^+$) in positive ion mode at 13.19 min at NCE 30 together with the EIC. Measurement of the pyruvate culture extract

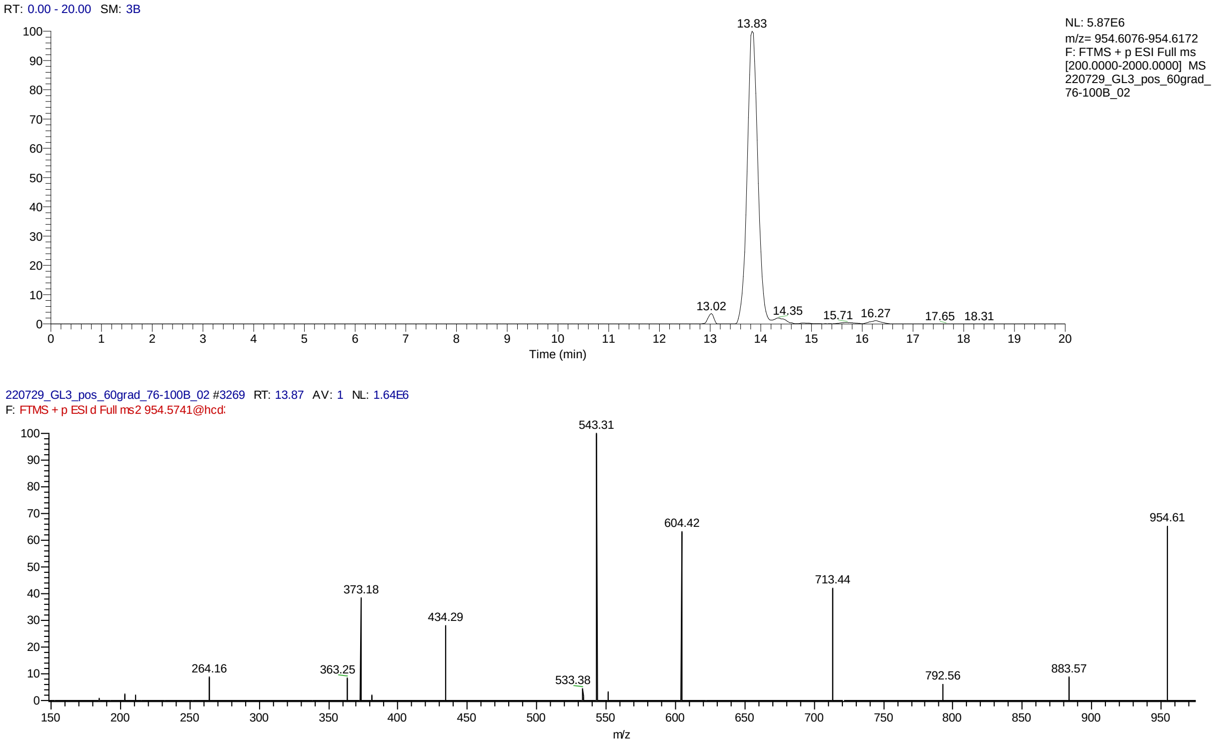

Fig. S25 MS/MS of Glc-40:0-Ala (*m/z* 954.6, $[M+Na]^+$) in positive ion mode at 13.87 min at NCE 30 together with the EIC. Measurement of the pyruvate culture extract

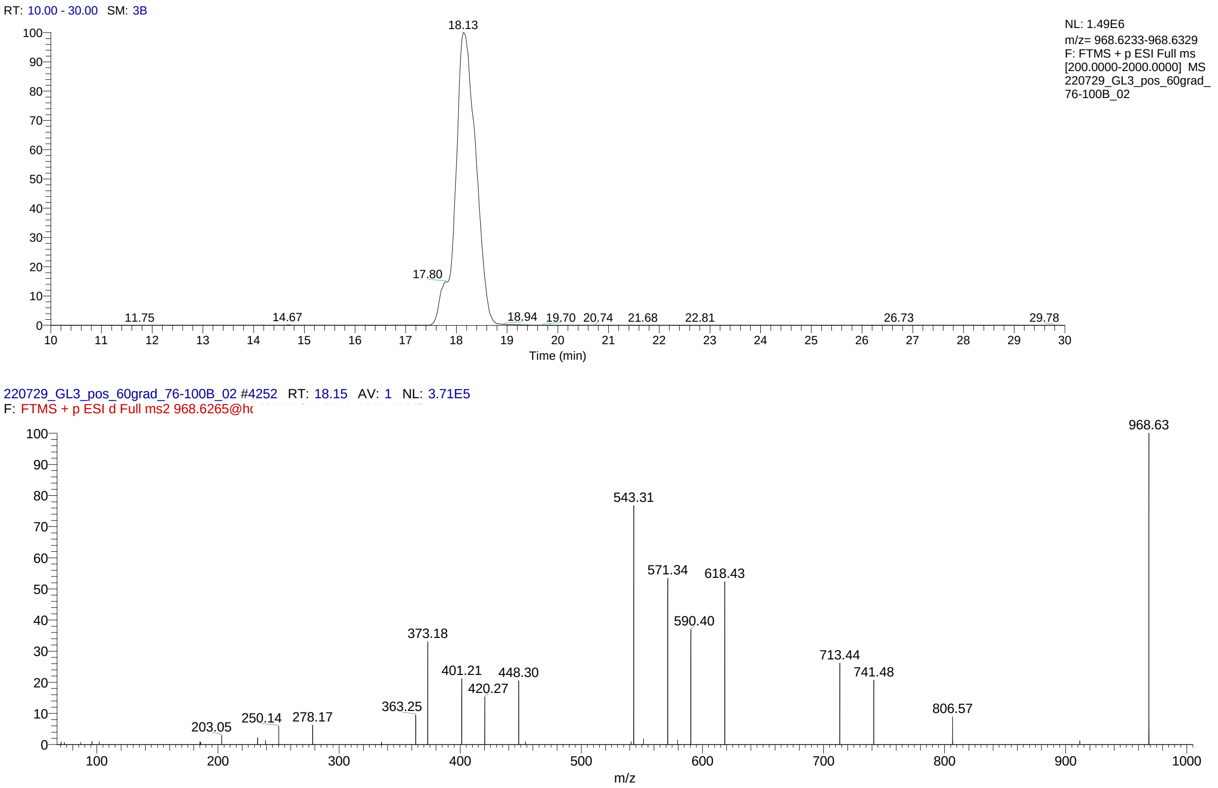

Fig. S26 MS/MS of Glc-42:0-Gly (*m/z* 968.6, $[M+Na]^+$) in positive ion mode at 18.15 min at NCE 30 together with the EIC. Measurement of the pyruvate culture extract

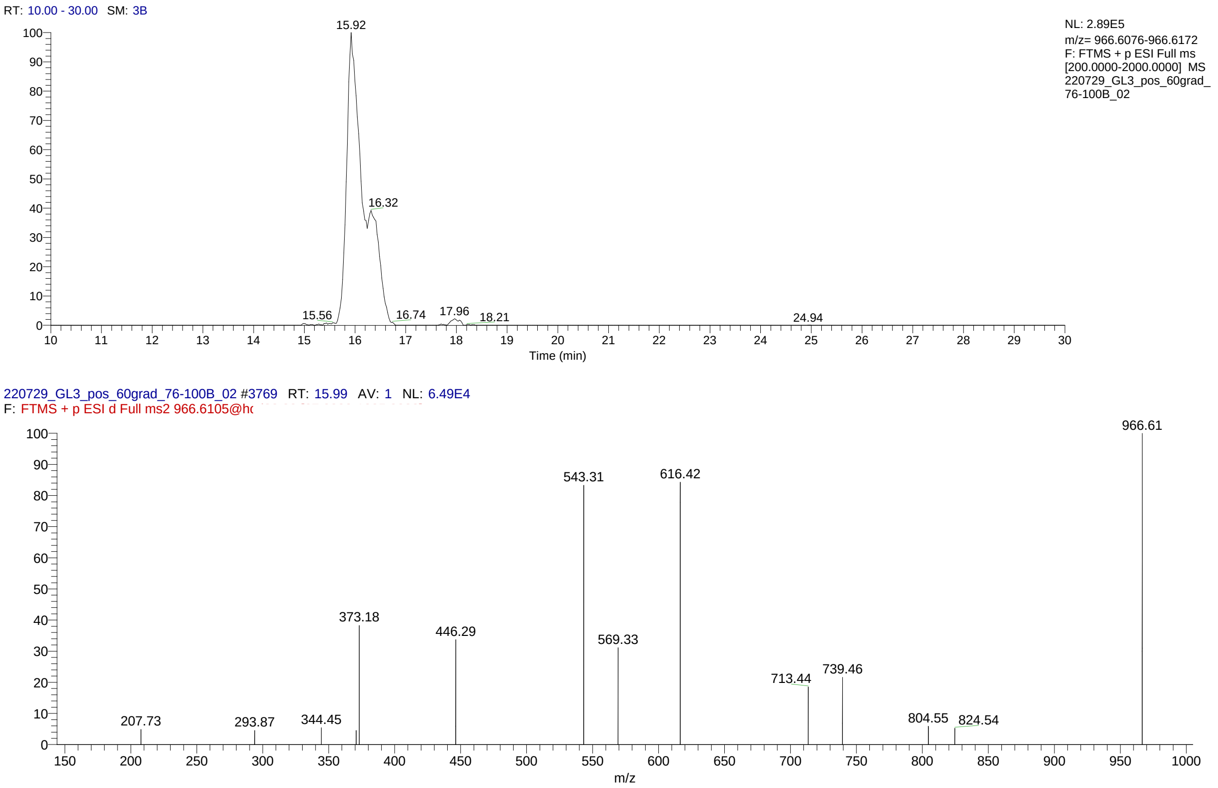

Fig. S27 MS/MS of Glc-42:1-Gly (*m/z* 966.6, $[M+Na]^+$) in positive ion mode at 15.99 min at NCE 30 together with the EIC. Measurement of the pyruvate culture extract

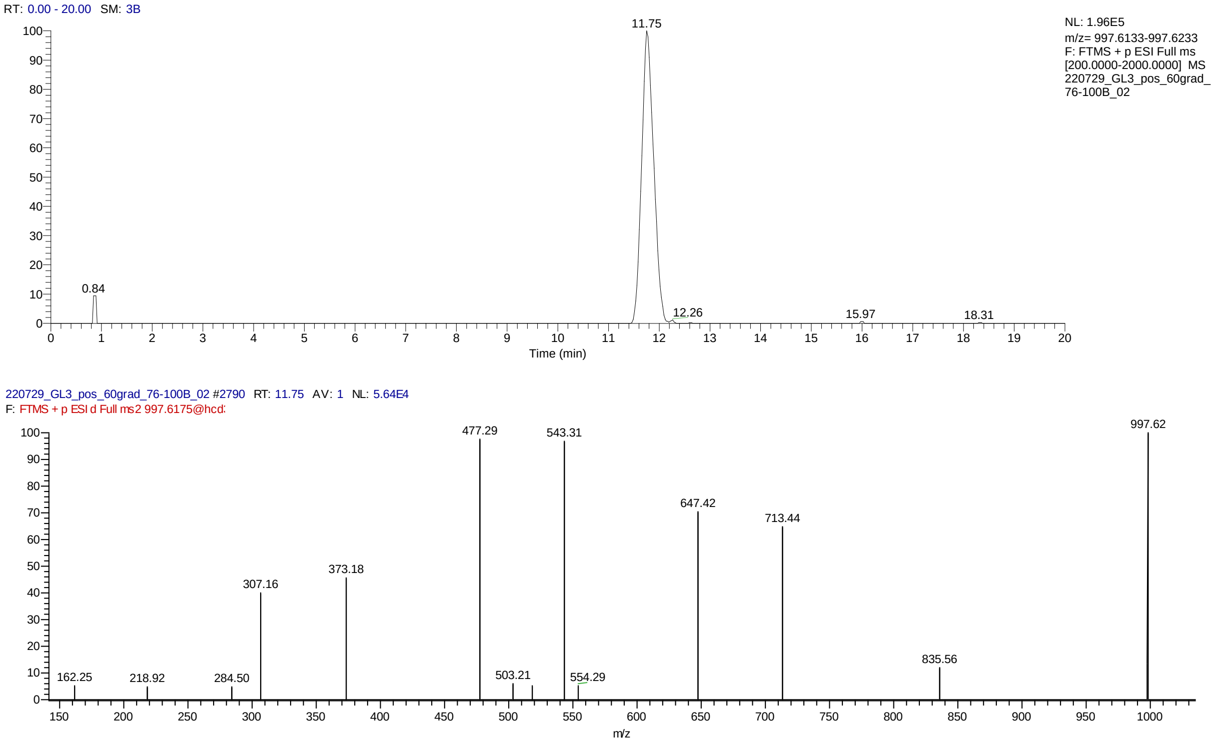

Fig. S28 MS/MS of Glc-40:0-Gly_2 (*m/z* 997.6, $[M+Na]^+$) in positive ion mode at 11.75 min at NCE 30 together with the EIC. Measurement of the pyruvate culture extract

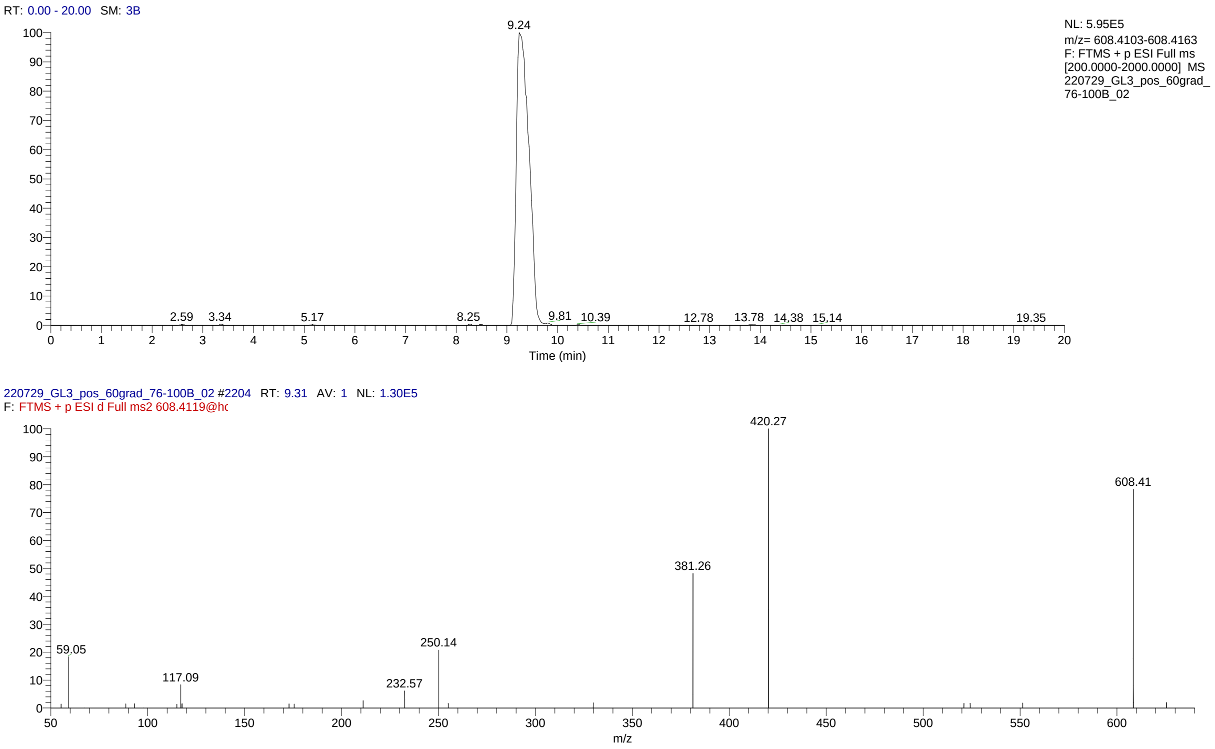

Fig. S29 MS/MS of 3×10:0-Gly (*m/z* 608.4, $[M+Na]^+$) in positive ion mode at 9.31 min at NCE 30 together with the EIC. Measurement of the pyruvate culture extract

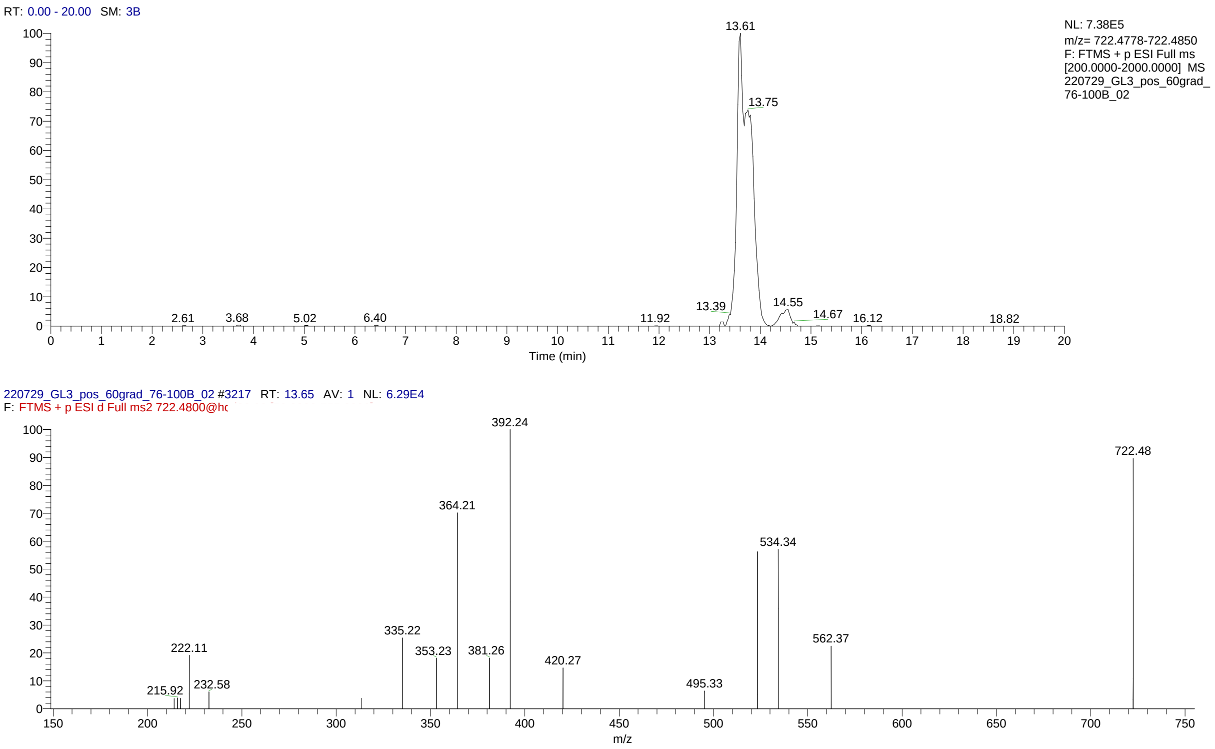

Fig. S30 MS/MS of 36:0-Gly (*m/z* 722.5, $[M+Na]^+$) in positive ion mode 13.65 min at NCE 30 together with the EIC. Measurement of the pyruvate culture extract

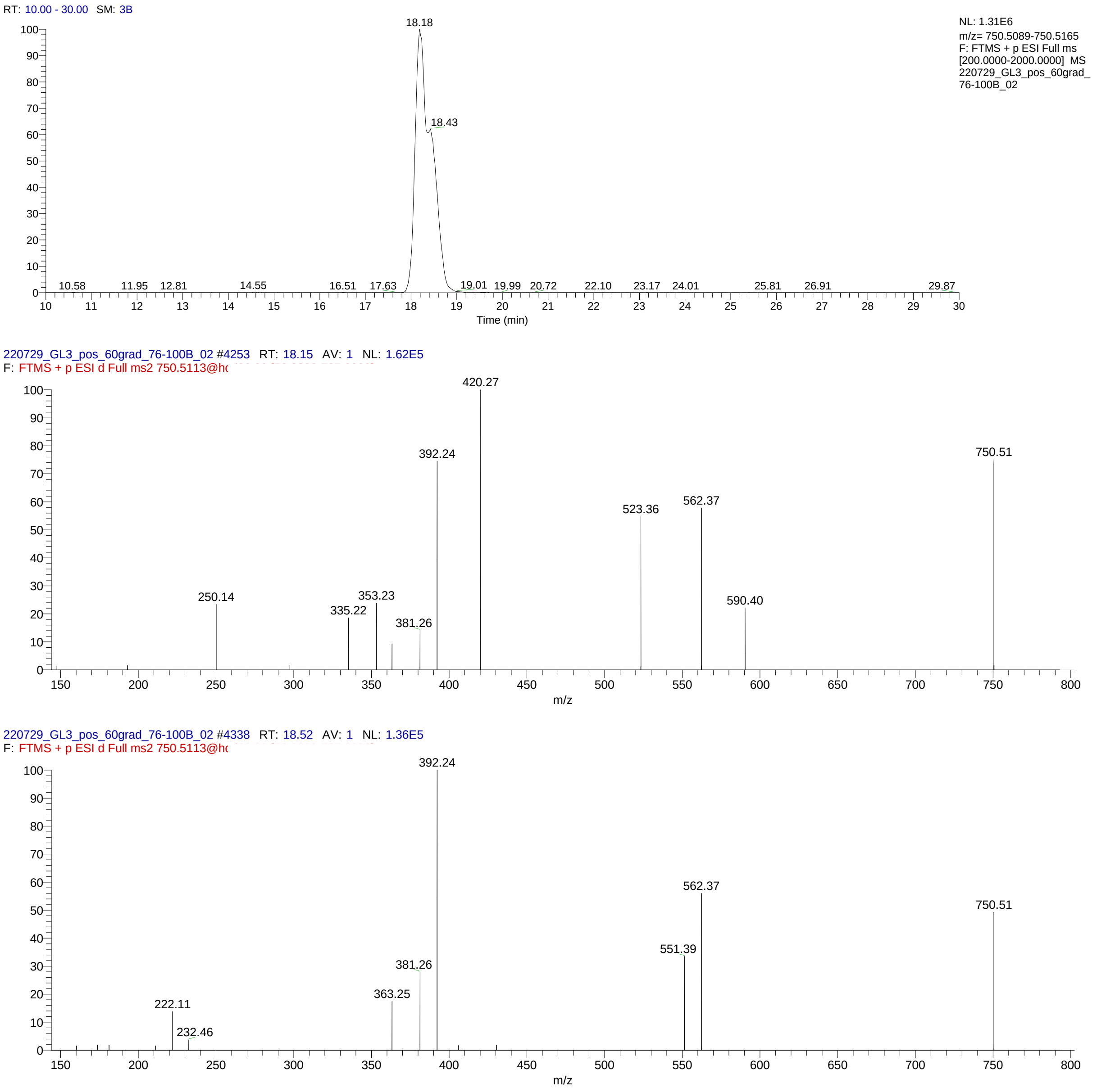

Fig. S31 MS/MS of 38:0-Gly (*m/z* 750.5, $[M+Na]^+$) in positive ion mode at 18.15 min and 18.52 min at NCE 30 together with the EIC. Measurement of the pyruvate culture extract

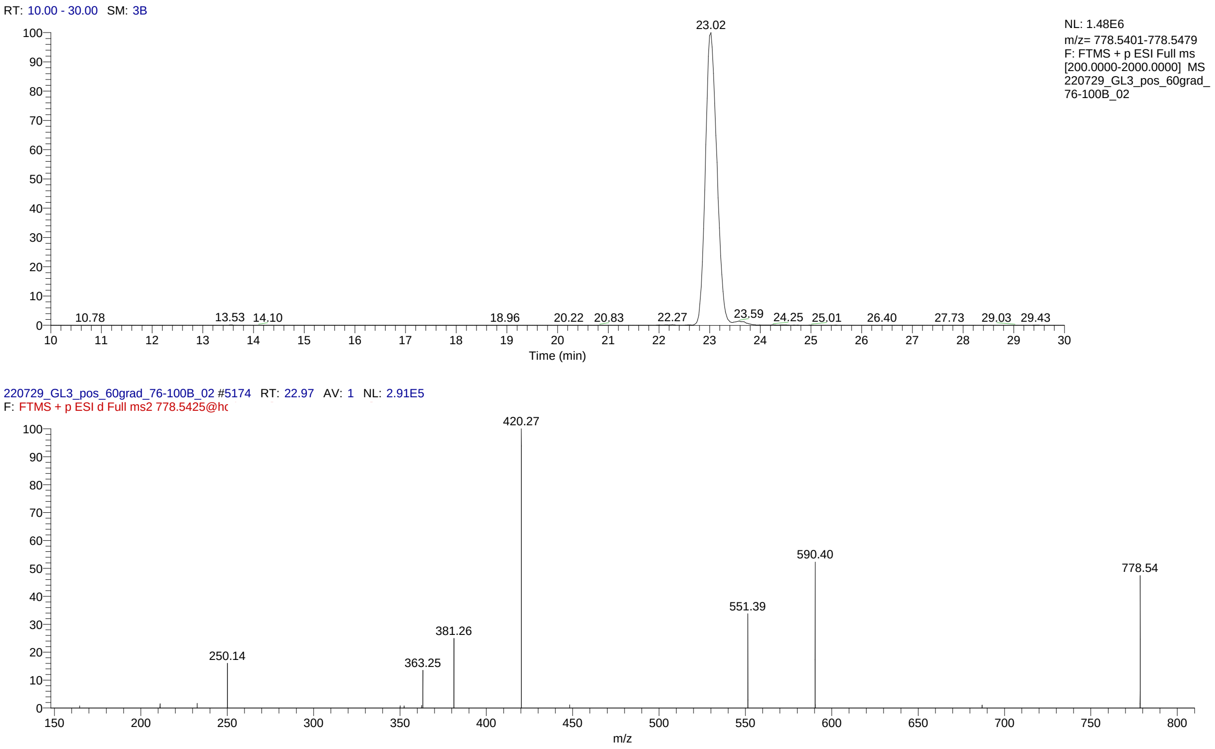

Fig. S32 MS/MS of 40:0-Gly (*m/z* 778.5, $[M+Na]^+$) in positive ion mode at 22.97 min at NCE 30 together with the EIC. Measurement of the pyruvate culture extract

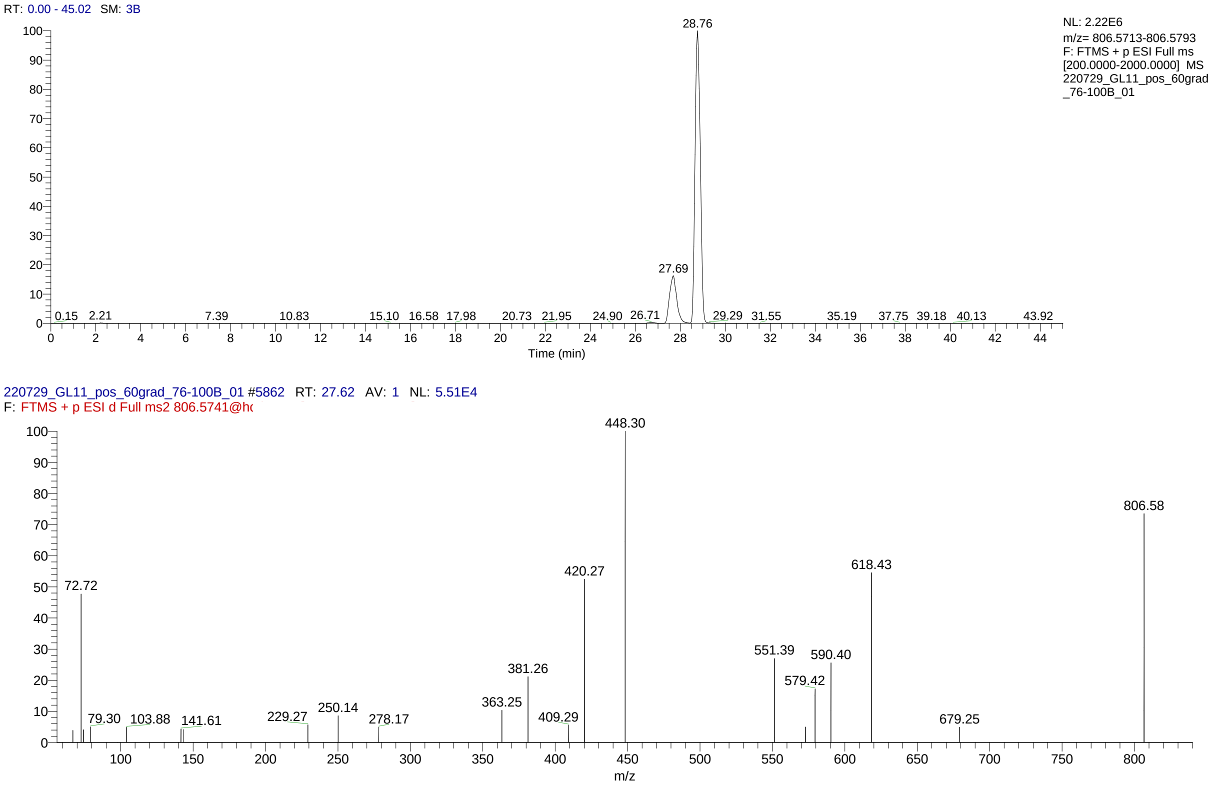

Fig. S33 MS/MS of 42:0-Gly (*m/z* 806.6, $[M+Na]^+$) in positive ion mode at 27.62 min at NCE 30 together with the EIC. Measurement of the pyruvate culture extract

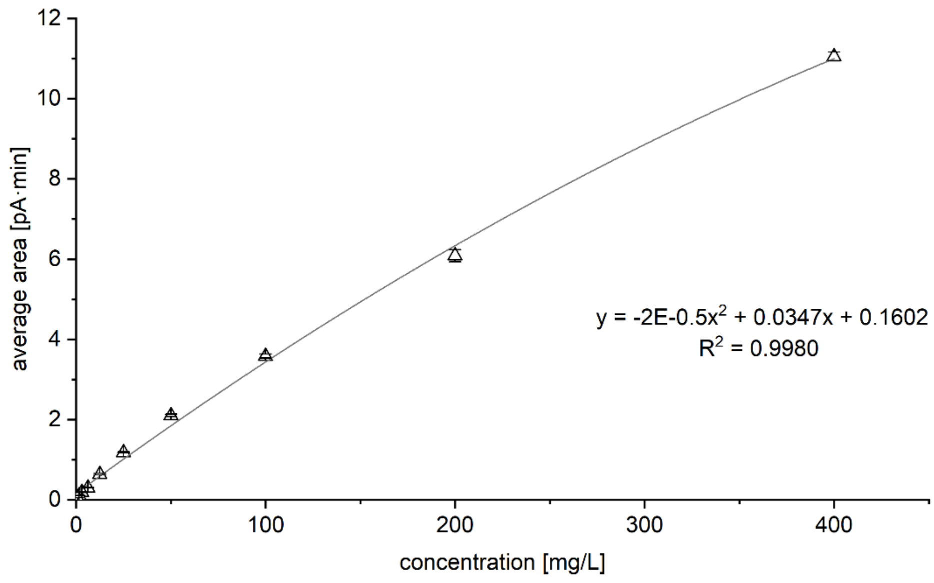

Fig. S34 Calibration curve for external calibration with 1-monoolein in the 0.8 to 400 mg/L concentration range (equivalent to 3.9 to 2000 ng on the column). A second-order polynomial fit is shown. Measurements were carried out in duplicate; the error bars indicate the range of the results obtained.
